# Transcriptomic analysis of the role of RasGEF1B circular RNA in the TLR4/LPS pathway

**DOI:** 10.1038/s41598-017-12550-w

**Published:** 2017-09-25

**Authors:** Wei Lun Ng, Georgi K. Marinov, Yoon-Ming Chin, Yat-Yuen Lim, Chee-Kwee Ea

**Affiliations:** 1 Institute of Biological Sciences, Faculty of Science, University of Malaya, Kuala Lumpur, 50603 Malaysia; 2 Department of Genetics, Stanford University School of Medicine, Stanford, CA 94305 United States; 3 Present Address: Department of Molecular Biology, University of Texas Southwestern Medical Center, Dallas, TX 75390-9148 United States

## Abstract

Circular RNAs (circRNAs) have recently emerged as a large class of novel non-coding RNA species. However, the detailed functional significance of the vast majority of them remains to be elucidated. Most functional characterization studies targeting circRNAs have been limited to resting cells, leaving their role in dynamic cellular responses to stimuli largely unexplored. In this study, we focus on the LPS-induced cytoplasmic circRNA, *mcircRasGEF1B*, and combine targeted *mcircRasGEF1B* depletion with high-throughput transcriptomic analysis to gain insight into its function during the cellular response to LPS stimulation. We show that knockdown of *mcircRasGEF1B* results in altered expression of a wide array of genes. Pathway analysis revealed an overall enrichment of genes involved in cell cycle progression, mitotic division, active metabolism, and of particular interest, NF-κB, LPS signaling pathways, and macrophage activation. These findings expand the set of functionally characterized circRNAs and support the regulatory role of *mcircRasGEF1B* in immune response during macrophage activation and protection against microbial infections.

## Introduction

Circular RNAs (circRNAs) are a unique class of endogenous noncoding RNAs formed by the backsplicing of linear transcripts into a covalently closed circular molecule. Although some circRNAs were initially identified decades ago, they were long considered to be mere alternative splicing by-products of little biological importance^1,2^. However, primarily thanks to the advent of high-throughput sequencing technologies over the last decade, this perception has now been changed by multiple reports showing that a large number of circRNAs are generated by thousands of loci in the human, mouse, and other genomes, in cell-type specific manner^3–6^, and that some of these circRNAs are in fact functional. Several algorithms have been developed for the identification of circRNAs to elucidate their functions^7–9^.

The functions of circRNAs appear to be mostly manifested via post-transcriptional regulatory mechanism. CircRNAs can play the role of miRNA sponges^3,5,10–15^, by sequestering miRNAs through base pair complementarity, thus keeping them away from their mRNA targets. This is the mechanism of action that has received the most attention so far. However, the potential role of circRNAs as miRNA sponges seems to be limited by the fact that most of them have very few binding sites for specific miRNAs, and indeed, additional mechanisms of circRNA action have been discovered more recently. In addition to serving as miRNA sponges, circRNAs can also play the role of RNA-binding protein decoys, they can regulate alternative splicing, and finally, some may have direct effects on transcription^16,17^. Recent evidence also suggests the translation ability of circRNA via splicing-dependent, cap-independent manner^18^.

Despite these advances, the number of functionally characterized circRNAs remains very low in relative to the total number of circRNAs that are generated in mammalian cells, and their mechanisms of action are far from being fully elucidated. One neglected aspect of circRNA biology is the role that circRNAs might play in the regulation of dynamic cellular responses to external stimuli, as most previous studies have focused their efforts on studying circRNAs in resting cells.

Lipopolysaccharide (LPS) is a major component of the outer membrane of Gram-negative bacteria. Upon binding of LPS to the toll-like receptor 4 (TLR4), macrophages are activated leading to a myriad of biological responses that results in the induction of innate immune response along with the release of different immunomodulating molecules including tumour necrosis factor-alpha (TNFα), interleukin 1 (IL-1), IL-6^19,20^, macrophage inflammatory proteins (MIP) and IP10^21^. The release of these cytokines and chemokines is important for pro-apoptotic activity of the activated macrophage and attracting neutrophils, natural killer cells and activated T-cells for host-cell defense^21^.

Previously, we identified a circRNA, *mcircRasGEF1B*, the expression of which is induced upon lipopolysaccharide (LPS) stimulation and which appears to regulate *ICAM-1* transcript stability and therefore its protein levels in the mouse macrophages^22^. LPS stimulation, through the TLR4/LPS pathway, triggers the activation of a large orchestrated transcriptional network involving a wide array of NF-κB responsive genes, and is thus an ideal system to address in details the significance of circRNA species such as *mcircRasGEF1B* in cellular responses to external stimuli.

To this end, we depleted *mcircRasGEF1B* in mouse macrophages and studied the effects of its knockdown on transcriptome dynamics during LPS response using RNA sequencing (RNA-seq). Analysis of the RNA-seq data revealed that depletion of *mcircRasGEF1B* results in the misregulation of hundreds of genes, enriched for functional categories involved in cell cycle progression, mitotic division, metabolic activity, NF-κB and LPS signaling pathways, and macrophage activation. These findings confirm that *mcircRasGEF1B* plays a functional role in the process of cellular response to LPS stimulation.

## Results and Discussion

### Characterizing the effects of *mcircRasGEF1B* knockdown on the transcriptome during LPS stimulation

Previous study demonstrated that knocking down *mcircRasGEF1B* reduces transcript and protein levels of the LPS-induced *ICAM-1* gene by destabilizing its mature mRNA products. However, the question of to what extend *mcircRasGEF1B* is an important regulator of the inflammatory network remains open. To address this question, we characterized the genome-wide gene expression dynamics upon activation of the TLR4/LPS pathway in control and *mcircRasGEF1B*-deficient backgrounds.

**Figure 1:**
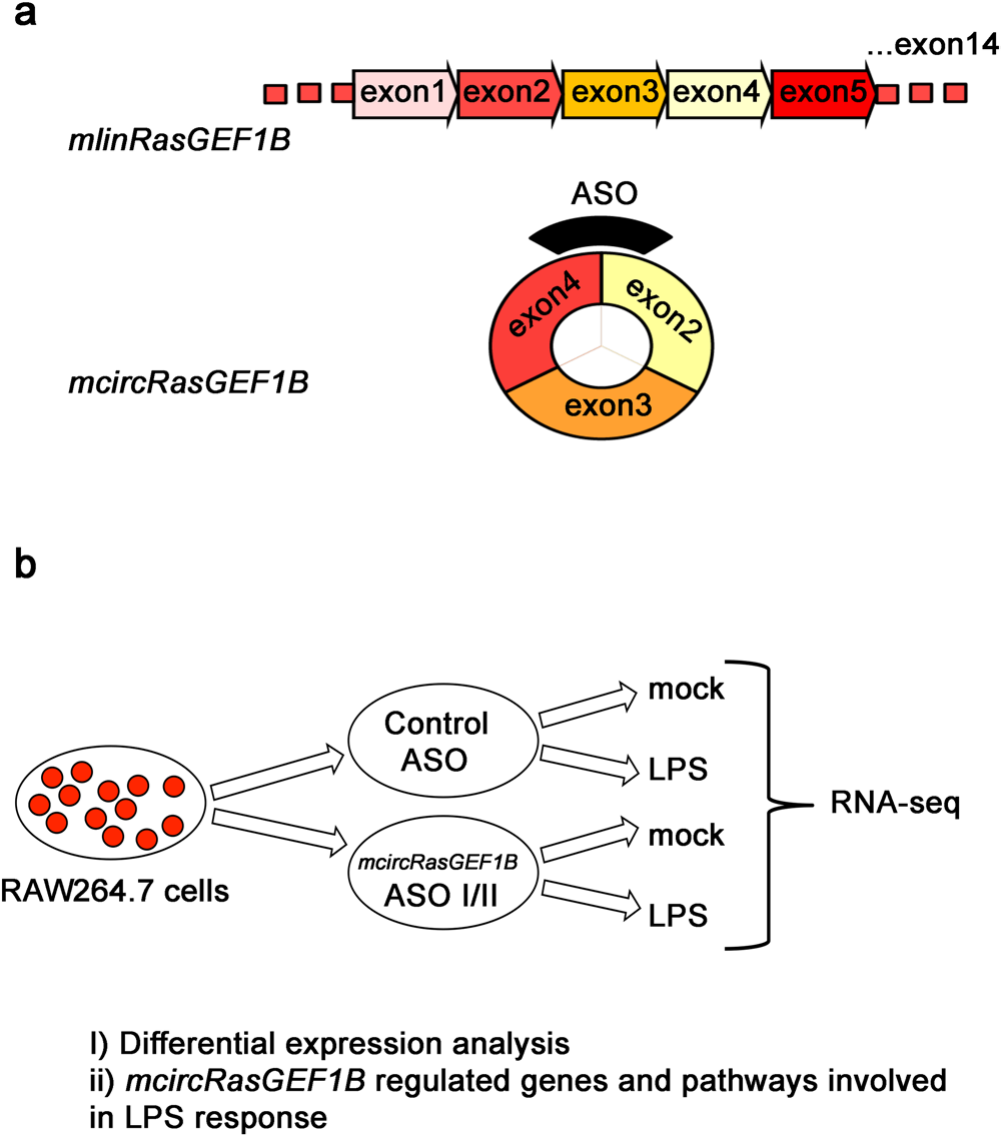
Characterization of the role of *mcircRasGEF1B* in LPS response. (**a**) *mcircRasGEF1B* is produced by the *RasGEF1B* locus in mouse through backsplicing. Antisense oligos (ASO) were designed specifically targeting the backsplice junction for the purpose of depleting *mcircRasGEF1B*. (**b**) RAW264.7 cells were treated with the *mcircRasGEF1B* targeting ASOI and ASOII oligos as well as with a control oligo, then subjected to LPS treatment (n = 3). Gene expression changes were then characterized at the global level using RNA-seq, thus identifying the genes and pathways that appear to be regulated by *mcircRasGEF1B*.

To determine how knockdown of *mcircRasGEF1B* alters the transcriptomic profile of murine macrophage upon LPS stimulation, we knocked down the expression of *mcircRasGEF1B* in RAW264.7 cells using two different antisense oligonucleotides (ASOs), ASO I and II, both of them targeting the back-splice junction unique to *mcircRasGEF1B* (Fig. 1). A sense-strand version of ASO I was used as a control^22^. We observed robust knockdown effciency, with ASO I reducing *mcircRasGEF1B* levels by 76% and ASO II depleting *mcircRasGEF1B* by 85% (Supplementary Fig. S1a). In agreement with our previous findings, we also observed reduction of *ICAM-1* expression in both ASO I, and ASO II-treated cells (Supplementary Fig. S1b).

We collected total RNA from three biological replicates of RAW264.7 cells of all three (control, ASO I and ASO II) groups, with and without LPS stimulation, and carried out RNA-seq experiments after rRNA removal. After mapping reads to the genome (Supplementary Table S1), quantification at the gene level, and extraction of read counts for each gene, genes differentially expressed upon LPS stimulation in each group, and genes differentially expressed between control and *mcircRasGEF1B*-knockdown cells were identified using DESeq2^23^ (see the Methods section for details).

### Gene expression changes during LPS response

**Figure 2:**
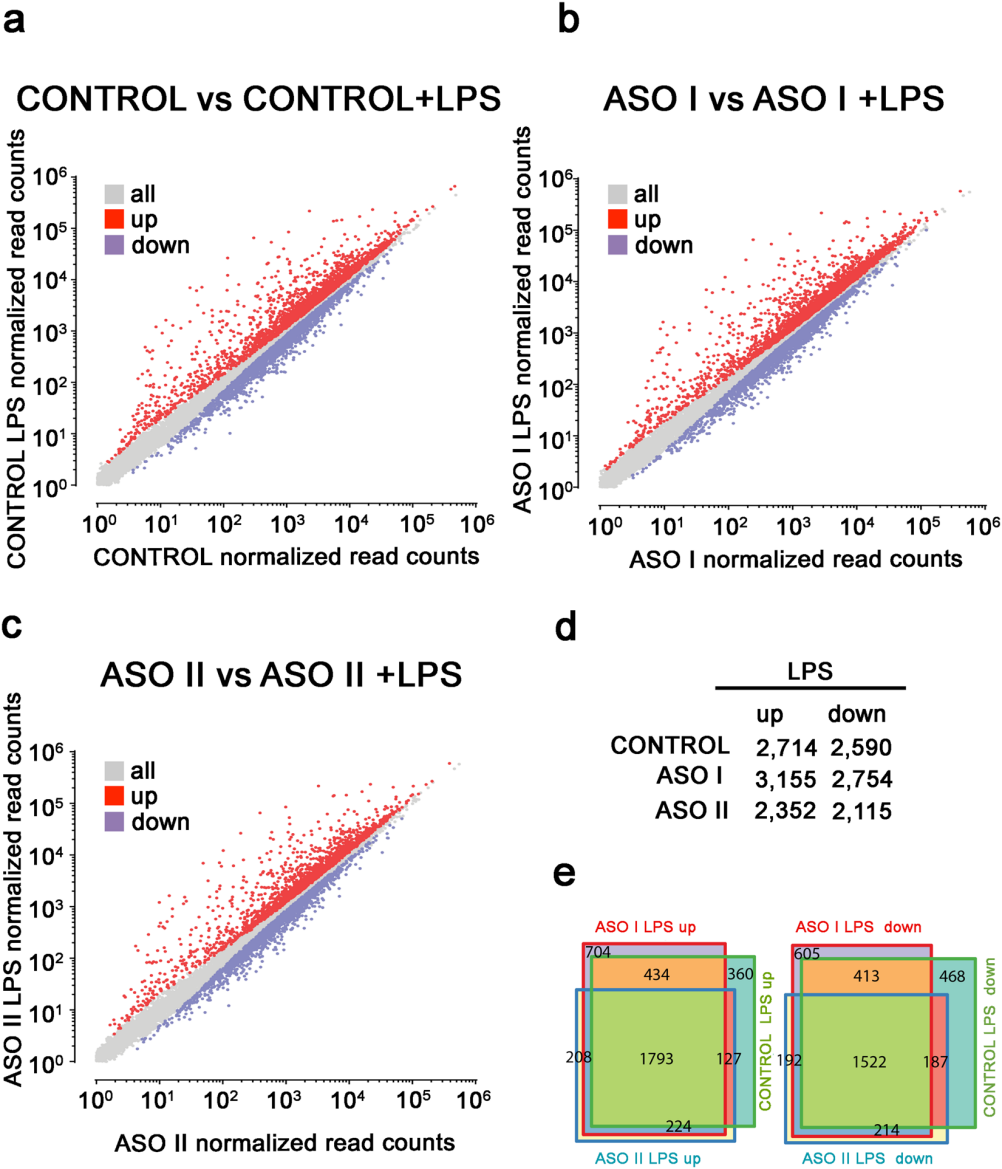
Gene expression changes upon LPS stimulation. (**a**–**c**) Scatter plots show gene expression changes relative to resting cells after LPS treatment in (**a**) control cells, (**b**) ASO I-treated cells; and (**c**) ASO II-treated cells. (**d**) Number of differentially expressed genes upon LPS stimulation in all conditions. (**e**) Overlap between the sets of differentially expressed genes upon LPS treatment in control, ASO I- and ASO II- treated cells.

After correcting for multiple hypothesis testing and setting an adjusted *p*-value threshold of 0.05, we identified 2,714 upregulated, and 2,590 downregulated genes in control cells after LPS stimulation (Fig. 2a and d). In the ASO I-transfected cells, 3,155 and 2,754 genes were upregulated and downregulated, respectively (Fig. 2b and d), while 2,352 and 2,115 genes were upregulated and downregulated in ASO II-treated cells (Fig. 2c and d). To confirm the specificity of the observed transcriptional response, we examined the top upregulated genes upon LPS stimulation in control cells, and found that most are immune-related genes such as *IL23a*, *CXCL10, CCL5*, *IL6*, *IL1B*, and *IFNB1* (Supplementary Fig. S2). We also compared the LPS-responsive genes between control, ASO I-, and ASO II-treated cells, and observed that 1,793 upegulated and 1,522 downregulated genes were common to all three conditions (Fig. 2e). However, a number of genes were only up or downregulated in control or in control and ASO I-treated cells, with ASO II-treated cells exhibiting the fewest LPS-responsive genes. As noted above and in Supplementary Fig. S1, ASO I decreased *mcircRasGEF1B* levels by 76% compared to the 85% knockdown observed in ASO II treatments. These observations are therefore consistent with the greater depletion of *mcircRasGEF1B* in ASO II-treated cells affecting LPS response to a greater extent than the more moderate knockdown in ASO I-treated cells.

### Gene expression changes upon *mcircRasGEF1B* depletion

**Figure 3:**
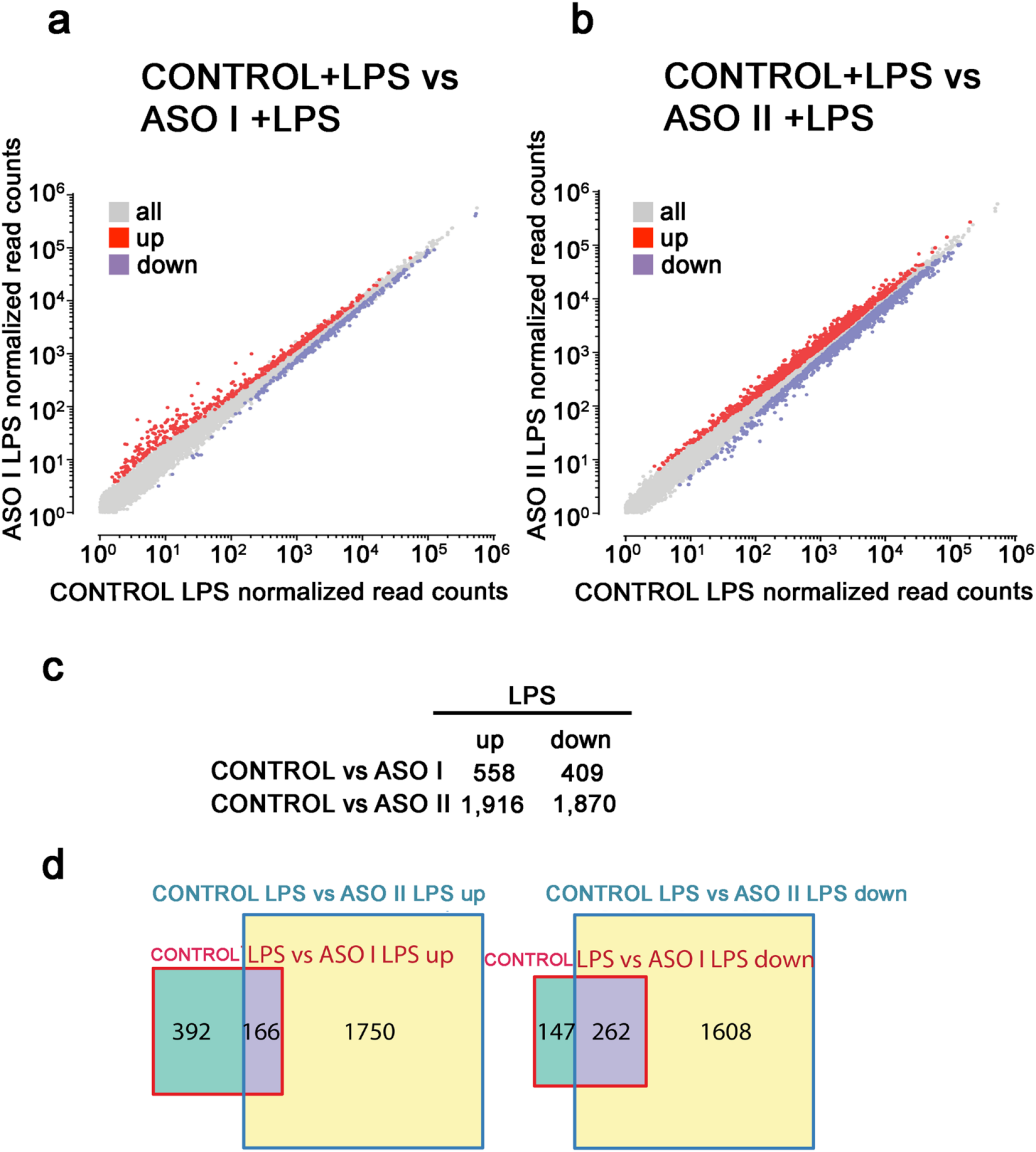
Gene expression changes upon *mcircRasGEF1B* depletion. (**a** and **b**) Scatter plots show gene expression changes in (**a**) LPS-stimulated ASOI-treated cells; (**b**) LPS-stimulated ASO II-treated cells; relative to LPS-stimulated control cells. (**c**) Number of differentially expressed genes in ASOI- or ASO II-treated, and LPS-stimulated cells. (**d**) Overlap between differentially expressed genes in ASO I- or ASO II-treated, and LPS-stimulated cells.

To directly examine the role of *mcircRasGEF1B* in the cellular response to TLR4/LPS pathway activation, we compared differentially expressed genes between ASO I-treated, ASO II-treated and control cells upon LPS stimulation (Fig. 3). We observed 558 upregulated and 409 downregulated genes after LPS stimulation in ASO I-treated cells relative to control cells (Fig. 3a and c). The transcriptome profiles of ASO II-treated cells were considerably more different, with 1,916 upregulated and 1,870 downregulated genes (Fig. 3b and c), again consistent with the higher efficiency of ASO II-mediated *mcircRasGEF1B* knockdown. We also compared the LPS-responsive genes between ASO I- and ASO-II-treated cells, and observed that 166 upregulated and 262 downregulated genes were common to both conditions (Fig. 3d). These results show that perturbation of *mcircRasGEF1B* affects the transcriptional or post-transcriptional regulation of hundreds to thousands of genes in response to LPS stimulation.

### Genes affected by *mcircRasGEF1B* depletion are enriched for functional categories related to LPS response

**Figure 4:**
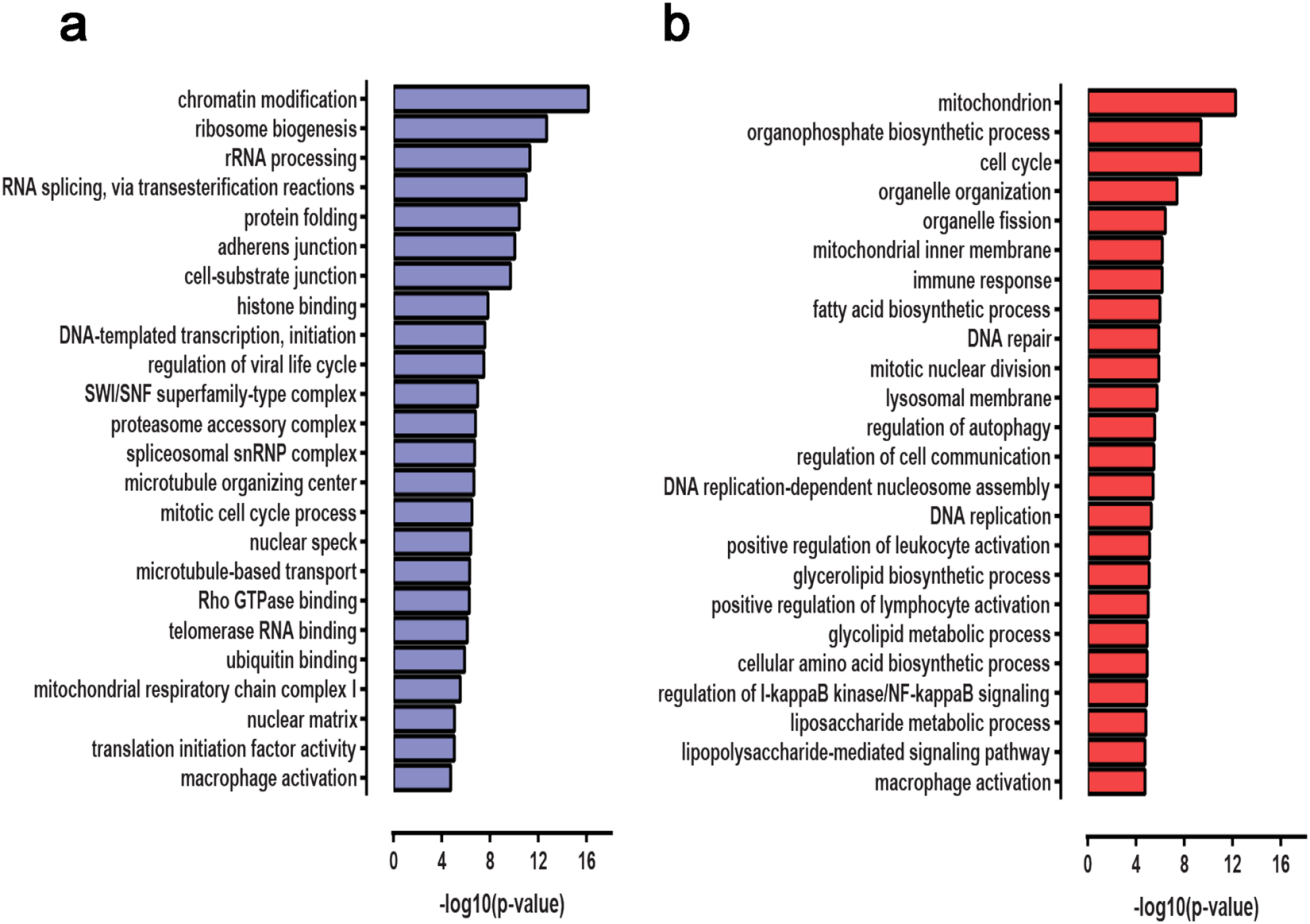
Functional categories enriched among differentially expressed genes in ASO II- and LPS-stimulated cells relative to control LPS-stimulated cells. Representative enriched functional categories are shown for (**a**) downregulated genes; (**b**) upregulated genes, with the x-axis indicating the statistical significance of the observed enrichment.

In order to understand the biological roles of genes misregulated upon *mcircRasGEF1B* depletion, we identified significantly enriched (*p* ≤ 0.05 after correcting for multiple hypothesis testing) gene ontology (GO) functional categories of genes in the sets of genes up- and downregulated relative to control in LPS-stimulated ASO-treated cells (Fig. 4; Supplementary Tables S2–S5). We focused on genes up- and downregulated in the ASO II background due to the higher magnitude of the effect of ASO II on the macrophage transcriptome profile. The GO analysis revealed that genes upregulated in *mcircRasGEF1B* knockdown cells are enriched for categories involved in metabolic activity, autophagy, DNA replication and mitotic division, macrophage activation too, and immune response, specifically the regulation of IκB/NFκB signaling and the LPS response pathway. The set of downregulated genes also revealed a number of coherent functional categories, specifically genes involved in chromatin remodeling, RNA splicing, cell adhesion, as well as, common with upregulated genes, mitochondrial respiratory function and macrophage activation. Detailed examination of the lists of downregulated genes corroborates these global observations. For example, among the top downregulated genes was *IFNB1*, a member of the type I interferons, which play key roles in the defense against viral infections and in the innate immune responses to pathogens; production of *IFNB1* is dependent on the LPS-induced TRIF-dependent pathway^24^. The LPS-mediated activation of RAW264.7 cells is known to be associated with the regulation of cell cycle progression^25^, and the NF-κB and TLR4/LPS signaling pathways are the mechanism through which LPS response is mediated, thus the observations of global misregulation of genes involved in these pathways underscore the functional importance of *mcircRasGEF1B* during LPS response.

## Conclusion

In this study, we identified a broad spectrum of genes involved in the cellular response to LPS activation whose proper expression dynamics is dependent on the LPS-inducible cytoplasmic circular RNA *mcircRasGEF1B*. We knocked down *mcircRasGEF1B* and studied the effects of its depletion on the transcriptome in resting and LPS-stimulated cells.

We observed that depletion of *mcircRasGEF1B* leads to the misregulation of a plethora of genes, among them functional modules involved in cell cycle progression, macrophage activation and LPS response signaling, cell adhesion and metabolic activity. Normal levels of *mcircRasGEF1B* thus appear to be important for the proper progression of macrophage activation and LPS signaling, and *mcircRasGEF1B* likely plays an important role in the process, confirming its functional significance. Further experiments should reveal in detail the precise mechanisms through which *mcircRasGEF1B* exercises its function.

## Materials and Methods

### Cell culture and reagents

RAW264.7 cells were cultured in Rosewell Park Memorial Institute medium (RPMI), supplemented with 10% FBS, 20 U/ml penicillin and 100 µg/ml streptomycin (GIBCO). LPS was purchased from Sigma.

### ASOs transfection

ASOs (Supplementary Table S6) were synthesized by IDT technologies. ASOs (20 nM) were transfected into RAW264.7 cells with the X-tremeGENE HP DNA (Roche) according to the manufacturer’s protocol. To maximize knockdown efficiency, ASO transfection was repeated 24 hours after the initial transfection. Cells were then treated with LPS for 2 hours.

### Quantitative RT-PCR

Total RNA was isolated with the Thermo Scientific GeneJET RNA Purification Kit. Complementary DNAs were synthesized and quantitative RT-PCR was performed with 2X SYBR Green PCR Master mix (Thermo Scientific) and run on a Bio-Rad Connect Real-Time PCR System. Expression levels of circular RNAs described in this study were measured by qPCR using gene specific divergent primers (Supplementary Table S6). The relative expression levels of circular versus linear isoforms were normalized to the *L32* gene.

### RNA extraction, library preparation, and sequencing

Total RNA was isolated with the Thermo Scientific GeneJET RNA Purification Kit. The RNA samples were checked for quality using Bio-Analyzer 2100 (Agilent Technologies, San Diego, CA, USA) and Qubit RNA assay kit. 1.5 μg of total RNA from each sample was used to prepare the library using ScriptSeq Complete Kit (Epicentre Inc, Madison, WI, USA) according to the manufacturer’s protocol.

### RNA-seq data processing and analysis

Except where otherwise indicated, all analysis were carried out using custom-written Python scripts.

Paired-end (2 × 75 bp) RNA-seq reads were aligned against the mm9 version of the mouse genome using TopHat2^26^ (version 2.0.8, run with Bowtie^27^ version 0.12.9) and the Ensembl 66 annotation with the following parameters:–no-discordant–no-mixed–read-realign-edit-dist 0–read-edit-dist 4–read-mismatches 4–min-segment-intron 10–min-coverage-intron 10. Read mapping statistics can be found in the Supplementary Table S1. Raw sequencing reads are available from the Gene Expression Omnibus under GEO accession number GSE99811.

Gene-level quantification in FPKM (Fragments Per Kilobase per Million mapped fragments) units was carried out using Cufflink^28^ (version 2.0.2).

For differential expression analysis, sequencing counts at the gene level were obtained using HTSeq^29^ (version 0.6.1p1). DESeq2^23^ was then used to identify differential expressed genes between different conditions. We note that one of the three replicates of unstimulated ASO II treated cells exhibited a globally discordant transcriptomic profile, and was accordingly excluded from the differential expression analysis.

Statistically enriched functional categories of genes were identified using FuncAssociate 2.0^30^.

## Electronic supplementary material

Supplementary materials
