# Supplementary material for: Transcriptomic analysis of the role of RasGEF1B circular RNA in the TLR4/LPS pathway
Source: Sci Rep. 2017 Sep 25;7:12227. doi: 10.1038/s41598-017-12550-w (PMC5612941; doi:10.1038/s41598-017-12550-w)
Supplement: Supplementary file 1 — Supplementary materials [file 41598_2017_12550_MOESM1_ESM.pdf]

# Supplementary Materials
# Transcriptomic analysis of the role of RasGEF1B circular RNA in the TLR4/LPS pathway

Wei Lun Ng[1], Georgi K Marinov[2], Yoon-Ming Chin[1], Yat-Yuen Lim[1,*], and Chee-Kwee Ea[1,‡,*]

[1]*Institute of Biological Sciences, Faculty of Science, University of Malaya, Kuala Lumpur, 50603, Malaysia*
[2]*Department of Genetics, Stanford University School of Medicine, Stanford, CA 94305, United States*
[‡]*Present Address: Department of Molecular Biology, University of Texas Southwestern Medical Center, Dallas, TX 75390-9148, United States*
[*]*Correspondence: Tel: 603–79677022ext2587; Fax: 603–79557727; E-mail: yatyuen.lim@um.edu.my; Chee-Kwee.Ea@utsouthwestern.edu*

## Supplemental Figures

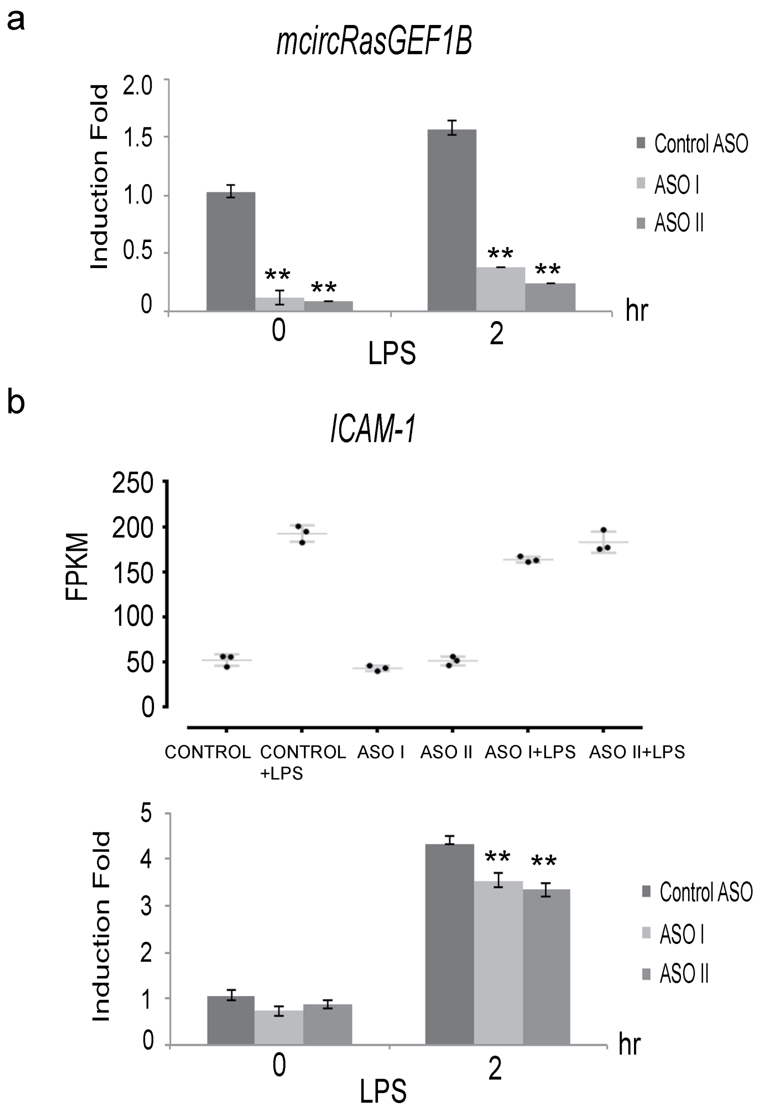


**Supplementary Figure 1: Efficiency and *ICAM-1* effect of *mcircRasGEF1B* knockdown** . (A) RAW264.7 cells were transfected with ASO I, ASO II and control ASO, and induced with LPS for 2 hours. The expression of *mcircRasGEF1B* was measured by qRT-PCR. (B) The expression level of *ICAM-1* in RNA-seq data (top); and qRT-PCR (bottom) was measured relative to *L32*. Error bars represent the variation range of triplicate experiments. (*, $p < 0.05$; **, $p < 0.01$).

| genes | $log_2FC$ | *p* -value |
|---|---|---|
| *Il23a* | 7.8938 | 0 |
| *Cxcl10* | 7.8371 | 0 |
| *Il1a* | 7.7641 | 0 |
| *Gbp5* | 7.3563 | 0 |
| *Ccl5* | 7.2981 | 0 |
| *Il6* | 7.1563 | 1.21E-156 |
| *Ccl22* | 7.0683 | 1.51E-138 |
| *Csf2* | 6.7174 | 0 |
| *Gm14047* | 6.7032 | 5.00E-135 |
| *Cxcl2* | 6.5618 | 0 |
| *Il1b* | 6.5494 | 2.19E-93 |
| *Gm8818* | 6.5230 | 4.24E-241 |
| *F3* | 6.4279 | 2.04E-121 |
| *Cxcl1* | 6.4135 | 5.00E-97 |
| *Edn1* | 6.3663 | 3.01E-185 |
| *Il27* | 6.3570 | 1.18E-247 |
| *Mir155* | 6.3131 | 7.98E-190 |
| *Ptgs2* | 6.3039 | 0 |
| *Sele* | 6.0683 | 5.69E-90 |
| *Ifnb1* | 5.8384 | 2.01E-89 |

**Supplementary Figure 2: Top 20 LPS-induced genes in control cells upon LPS stimulation**.

## Supplemental Tables

**Supplementary Table 1: Summary and read mapping statistics of RNA-seq samples used in this study.**

| Library | Unique | Unique Splices | Multi | Multi Splices |
|---|---|---|---|---|
| Control Rep1 | 4,001,553 | 1,576,031 | 272,043 | 18,472 |
| Control Rep2 | 3,981,124 | 1,517,314 | 301,195 | 19,716 |
| Control Rep3 | 3,997,278 | 1,481,999 | 393,086 | 22,969 |
| Control LPS-Rep1 | 4,085,432 | 1,590,647 | 397,261 | 23,080 |
| Control LPS-Rep2 | 4,554,974 | 1,686,258 | 410,300 | 24,515 |
| Control LPS-Rep3 | 4,337,367 | 1,512,642 | 352,840 | 20,430 |
| ASO1-Rep1 | 4,525,466 | 1,390,815 | 266,836 | 21,194 |
| ASO1-Rep2 | 4,823,690 | 1,430,735 | 287,056 | 24,248 |
| ASO1-Rep3 | 5,076,425 | 1,289,118 | 273,866 | 17,786 |
| ASO1-LPS-Rep1 | 4,478,738 | 1,297,073 | 292,012 | 18,822 |
| ASO1-LPS-Rep2 | 4,234,949 | 1,547,274 | 320,827 | 24,839 |
| ASO1-LPS-Rep3 | 4,184,707 | 1,290,558 | 265,492 | 17,471 |
| ASO2-Rep1 | 4,876,629 | 1,636,070 | 305,659 | 24,490 |
| ASO2-Rep2 | 4,620,523 | 1,535,349 | 274,289 | 21,648 |
| ASO2-Rep3 | 2,676,301 | 458,594 | 218,918 | 30,156 |
| ASO2-LPS-Rep1 | 4,213,403 | 1,452,831 | 268,594 | 23,455 |
| ASO2-LPS-Rep2 | 4,576,848 | 1,576,320 | 283,714 | 22,517 |
| ASO2-LPS-Rep3 | 4,040,789 | 1,303,825 | 251,539 | 19,734 |

**Supplementary Table 2: GO categories enriched ($p < 0.05$) in genes upregulated in ASO I LPS treated relative to Control LPS cells**

| N | X | LOD | P | P adj | attrib ID | attrib name |
|---|---|---|---|---|---|---|
| 3 | 3 | 2.44628582586375 | 1.47116730041235e-05 | 0.043 | GO:0008821 | crossover junction endodeoxyribonuclease activity |
| 9 | 54 | 0.924741122596219 | 6.02953623655649e-06 | 0.019 | GO:0000724 | double-strand break repair via homologous recombination |
| 9 | 54 | 0.924741122596219 | 6.02953623655649e-06 | 0.019 | GO:0000725 | recombinational repair |
| 9 | 57 | 0.896951797290675 | 9.55503105823617e-06 | 0.021 | GO:0051983 | regulation of chromosome segregation |
| 19 | 191 | 0.663761352736977 | 2.69770371720001e-07 | < 0.001 | GO:0090068 | positive regulation of cell cycle process |
| 14 | 142 | 0.659836036673664 | 1.1054858868697e-05 | 0.023 | GO:0006310 | DNA recombination |
| 24 | 264 | 0.621271446972535 | 4.21382738967266e-08 | < 0.001 | GO:0045787 | positive regulation of cell cycle |
| 40 | 476 | 0.59001014799207 | 1.45313539079928e-11 | < 0.001 | GO:1903047 | mitotic cell cycle process |
| 21 | 256 | 0.571331852274781 | 1.58753788296268e-06 | 0.002 | GO:0051302 | regulation of cell division |
| 21 | 261 | 0.562108519982127 | 2.16277609475737e-06 | 0.005 | GO:0007067 | mitotic nuclear division |
| 24 | 314 | 0.538247513173037 | 1.03276315271707e-06 | 0.002 | GO:0000280 | nuclear division |
| 54 | 739 | 0.529826988078727 | 9.42383894647487e-13 | < 0.001 | GO:0022402 | cell cycle process |
| 26 | 358 | 0.514477169624575 | 9.7218872942357e-07 | 0.001 | GO:0007346 | regulation of mitotic cell cycle |
| 32 | 450 | 0.506425008470359 | 8.86403957904801e-08 | < 0.001 | GO:0010564 | regulation of cell cycle process |
| 46 | 656 | 0.505874894710675 | 1.96048371895818e-10 | < 0.001 | GO:0007049 | cell cycle |
| 24 | 339 | 0.501896994914759 | 3.93321079070165e-06 | 0.008 | GO:0048285 | organelle fission |
| 27 | 389 | 0.493267540378836 | 1.44279392322174e-06 | 0.002 | GO:0051301 | cell division |
| 23 | 335 | 0.487194533980687 | 1.02809621512248e-05 | 0.023 | GO:0006281 | DNA repair |
| 48 | 740 | 0.469473444711532 | 1.05275272928914e-09 | < 0.001 | GO:0051726 | regulation of cell cycle |
| 33 | 537 | 0.43752477983833 | 1.52629114047526e-06 | 0.002 | GO:0006974 | cellular response to DNA damage stimulus |
| 33 | 567 | 0.411835514405273 | 4.88090561104063e-06 | 0.01 | GO:0006259 | DNA metabolic process |
| 53 | 1176 | 0.297397261140679 | 1.57157352707699e-05 | 0.044 | GO:0044430 | cytoskeletal part |
| 74 | 1682 | 0.293674789893107 | 6.98402629720436e-07 | < 0.001 | GO:0019899 | enzyme binding |
| 68 | 1612 | 0.270837946924649 | 8.55718138747276e-06 | 0.021 | GO:1902589 | single-organism organelle organization |
| 68 | 1630 | 0.265426030258141 | 1.22875441390808e-05 | 0.025 | GO:0043933 | macromolecular complex subunit organization |
| 84 | 2067 | 0.258433450625099 | 2.85274643098236e-06 | 0.006 | GO:0006996 | organelle organization |
| 264 | 7864 | 0.236405382901373 | 2.44250913729755e-10 | < 0.001 | GO:0044763 | single-organism cellular process |
| 96 | 2536 | 0.227243806495468 | 1.01362609561535e-05 | 0.023 | GO:0031325 | positive regulation of cellular metabolic process |
| 95 | 2511 | 0.226600733976012 | 1.16436738206674e-05 | 0.024 | GO:0010604 | positive regulation of macromolecule metabolic process |
| 109 | 2950 | 0.219537540108932 | 6.63281110263619e-06 | 0.019 | GO:0009893 | positive regulation of metabolic process |
| 137 | 3836 | 0.212072144103135 | 1.89389119904923e-06 | 0.005 | GO:0016043 | cellular component organization |
| 138 | 3898 | 0.207711380354226 | 2.76641260780079e-06 | 0.006 | GO:0071840 | cellular component organization or biogenesis |
| 169 | 5062 | 0.186759321408274 | 5.21140451900394e-06 | 0.016 | GO:0043167 | ion binding |
| 145 | 4289 | 0.185034269624009 | 1.75503894120654e-05 | 0.044 | GO:0048522 | positive regulation of cellular process |
| 316 | 10790 | 0.164077694786318 | 6.79970556531188e-06 | 0.019 | GO:0009987 | cellular process |
| 330 | 11392 | 0.162621689110978 | 8.89824451350892e-06 | 0.021 | GO:0005488 | binding |

**Supplementary Table 3: GO categories enriched ($p < 0.05$) in genes downregulated in ASO I LPS treated relative to Control LPS cells**

| N | X | LOD | P | P adj | attrib ID | attrib name |
|---|---|---|---|---|---|---|
| 5 | 16 | 1.42104395843672 | 6.84387029099494e-06 | 0.025 | GO:0005797 | Golgi medial cisterna |
| 5 | 18 | 1.35136898118145 | 1.3031150164929e-05 | 0.039 | GO:0042605 | peptide antigen binding |
| 6 | 30 | 1.1659501539057 | 1.35104997526562e-05 | 0.04 | GO:0035458 | cellular response to interferon-beta |
| 7 | 40 | 1.09312382161191 | 6.50049110815747e-06 | 0.021 | GO:0030593 | neutrophil chemotaxis |
| 7 | 42 | 1.06790118781991 | 9.11959605081532e-06 | 0.029 | GO:0001912 | positive regulation of leukocyte mediated cytotoxicity |
| 7 | 43 | 1.05581713121232 | 1.07254492225848e-05 | 0.031 | GO:1990266 | neutrophil migration |
| 8 | 50 | 1.04504446742259 | 2.85503673558999e-06 | 0.012 | GO:0071621 | granulocyte chemotaxis |
| 8 | 54 | 1.00590223383931 | 5.19619834085856e-06 | 0.015 | GO:0097530 | granulocyte migration |
| 11 | 88 | 0.917991174992937 | 5.20026130109419e-07 | 0.002 | GO:0030595 | leukocyte chemotaxis |
| 9 | 75 | 0.899532692642214 | 8.00018341052553e-06 | 0.025 | GO:0097529 | myeloid leukocyte migration |
| 10 | 84 | 0.894595612091377 | 2.70113278971453e-06 | 0.012 | GO:0061025 | membrane fusion |
| 9 | 80 | 0.867950424759251 | 1.36471648945705e-05 | 0.041 | GO:0002708 | positive regulation of lymphocyte mediated immunity |
| 11 | 99 | 0.860134153800073 | 1.71611169232395e-06 | 0.009 | GO:0002705 | positive regulation of leukocyte mediated immunity |
| 9 | 83 | 0.850041460392407 | 1.84464618390118e-05 | 0.05 | GO:0002824 | positive regulation of adaptive immune response based on somatic recombination of immune receptors built from immunoglobulin superfamily domains |
| 13 | 131 | 0.804597504513319 | 7.34346456478762e-07 | 0.003 | GO:0060326 | cell chemotaxis |
| 10 | 102 | 0.800257584681375 | 1.56382515194195e-05 | 0.042 | GO:0044448 | cell cortex part |
| 15 | 163 | 0.768200141509147 | 2.78602860753135e-07 | < 0.001 | GO:0005765 | lysosomal membrane |
| 15 | 163 | 0.768200141509147 | 2.78602860753135e-07 | < 0.001 | GO:0098852 | lytic vacuole membrane |
| 12 | 133 | 0.759160259493564 | 5.33040116785371e-06 | 0.015 | GO:0098858 | actin-based cell projection |
| 15 | 167 | 0.756578165700815 | 3.81670064893057e-07 | < 0.001 | GO:0002699 | positive regulation of immune effector process |
| 20 | 224 | 0.755107662216777 | 4.85739385134027e-09 | < 0.001 | GO:0007159 | leukocyte cell-cell adhesion |
| 17 | 190 | 0.755055042274383 | 6.76826378450233e-08 | < 0.001 | GO:0042110 | T cell activation |
| 17 | 191 | 0.752539422753393 | 7.30874827634445e-08 | < 0.001 | GO:0070489 | T cell aggregation |
| 17 | 193 | 0.747550806947227 | 8.5097066900818e-08 | < 0.001 | GO:0071593 | lymphocyte aggregation |
| 14 | 159 | 0.747056565058295 | 1.18568874658401e-06 | 0.005 | GO:0002703 | regulation of leukocyte mediated immunity |
| 17 | 199 | 0.73291461436296 | 1.3274508408242e-07 | < 0.001 | GO:0070486 | leukocyte aggregation |
| 15 | 178 | 0.726114068144416 | 8.65334376408855e-07 | 0.003 | GO:0005774 | vacuolar membrane |
| 18 | 215 | 0.723559566877548 | 7.7182844448673e-08 | < 0.001 | GO:0050730 | regulation of peptidyl-tyrosine phosphorylation |
| 16 | 194 | 0.715955956122226 | 5.00432694130978e-07 | < 0.001 | GO:0044437 | vacuolar part |
| 20 | 247 | 0.708366362405924 | 2.55550923471116e-08 | < 0.001 | GO:0034109 | homotypic cell-cell adhesion |
| 23 | 310 | 0.668206035346193 | 1.1968910115784e-08 | < 0.001 | GO:0000323 | lytic vacuole |
| 23 | 310 | 0.668206035346193 | 1.1968910115784e-08 | < 0.001 | GO:0005764 | lysosome |
| 18 | 257 | 0.638993452038462 | 1.0875318262984e-06 | 0.004 | GO:0050867 | positive regulation of cell activation |
| 15 | 215 | 0.636789492236678 | 8.95524236607567e-06 | 0.027 | GO:0051251 | positive regulation of lymphocyte activation |
| 24 | 353 | 0.627383634598059 | 3.09158732541079e-08 | < 0.001 | GO:0005773 | vacuole |
| 22 | 335 | 0.610081581718912 | 2.13211487925122e-07 | < 0.001 | GO:0050778 | positive regulation of immune response |
| 16 | 245 | 0.605807086713058 | 1.04280938165579e-05 | 0.03 | GO:0002696 | positive regulation of leukocyte activation |
| 17 | 263 | 0.601099535275709 | 6.35017590917767e-06 | 0.021 | GO:0032103 | positive regulation of response to external stimulus |
| 24 | 375 | 0.598877755136437 | 9.63525337514918e-08 | < 0.001 | GO:0016337 | single organismal cell-cell adhesion |
| 24 | 381 | 0.591408061713959 | 1.29317765547165e-07 | < 0.001 | GO:0002697 | regulation of immune effector process |
| 28 | 465 | 0.572337031511981 | 2.98417343948023e-08 | < 0.001 | GO:0050776 | regulation of immune response |
| 19 | 315 | 0.569124899011258 | 5.00312786359466e-06 | 0.014 | GO:0046649 | lymphocyte activation |
| 24 | 401 | 0.567370327514954 | 3.29803295132516e-07 | < 0.001 | GO:0098602 | single organism cell adhesion |
| 22 | 383 | 0.547259749977934 | 2.01544906510633e-06 | 0.01 | GO:0045321 | leukocyte activation |
| 60 | 1114 | 0.542872596367803 | 2.69647741842724e-14 | < 0.001 | GO:0002376 | immune system process |
| 123 | 2586 | 0.54100893201464 | 5.40566902184592e-25 | < 0.001 | GO:0070062 | extracellular exosome |
| 123 | 2588 | 0.540612604788522 | 5.792825693383e-25 | < 0.001 | GO:0065010 | extracellular membrane-bounded organelle |
| 19 | 335 | 0.540380160804644 | 1.20070895066237e-05 | 0.032 | GO:0001819 | positive regulation of cytokine production |
| 36 | 647 | 0.540117683820596 | 2.4428755749179e-09 | < 0.001 | GO:0002684 | positive regulation of immune system process |
| 123 | 2599 | 0.538437821696366 | 8.46249028486359e-25 | < 0.001 | GO:1903561 | extracellular vesicle |
| 123 | 2601 | 0.538043314957782 | 9.06389683502836e-25 | < 0.001 | GO:0043230 | extracellular organelle |
| 24 | 436 | 0.528168014272297 | 1.46264229948591e-06 | 0.007 | GO:0001775 | cell activation |
| 28 | 530 | 0.510883274945954 | 4.42537179230634e-07 | < 0.001 | GO:0048471 | perinuclear region of cytoplasm |
| 25 | 472 | 0.510607568102062 | 1.76788009963909e-06 | 0.009 | GO:0098609 | cell-cell adhesion |
| 132 | 3020 | 0.50692193539344 | 1.38144517673227e-23 | < 0.001 | GO:0031988 | membrane-bounded vesicle |
| 142 | 3443 | 0.487118682049634 | 3.68366600894311e-23 | < 0.001 | GO:0044421 | extracellular region part |
| 22 | 442 | 0.480430160782588 | 1.94230678329956e-05 | 0.05 | GO:0050865 | regulation of cell activation |
| 24 | 485 | 0.478444944936827 | 8.99044036297815e-06 | 0.027 | GO:0061024 | membrane organization |
| 137 | 3344 | 0.478226981443803 | 6.06275631257l2e-22 | < 0.001 | GO:0031982 | vesicle |
| 36 | 742 | 0.475491619129343 | 8.10813483166113e-08 | < 0.001 | GO:0006952 | defense response |
| 25 | 509 | 0.475369382599821 | 6.65354152884859e-06 | 0.021 | GO:0031347 | regulation of defense response |
| 25 | 517 | 0.468097317075596 | 8.69486140497124e-06 | 0.025 | GO:0098805 | whole membrane |
| 24 | 507 | 0.457781561031436 | 1.86222478239083e-05 | 0.05 | GO:0001817 | regulation of cytokine production |
| 26 | 554 | 0.454579201399188 | 9.72881009669264e-06 | 0.03 | GO:0006955 | immune response |
| 26 | 563 | 0.447065793027792 | 1.28782505581973e-05 | 0.037 | GO:0009986 | cell surface |
| 28 | 620 | 0.437530467695154 | 9.03168873357259e-06 | 0.028 | GO:0005768 | endosome |
| 47 | 1096 | 0.424071861922703 | 3.46654240256073e-08 | < 0.001 | GO:0002682 | regulation of immune system process |
| 31 | 717 | 0.4185931341424 | 7.13731393955370e-06 | 0.025 | GO:0016192 | vesicle-mediated transport |
| 199 | 6305 | 0.401125592094758 | 1.16693652223884e-19 | < 0.001 | GO:0044444 | cytoplasmic part |
| 322 | 13700 | 0.393396869499844 | 7.84418067107261e-16 | < 0.001 | GO:0044464 | cell part |
| 33 | 808 | 0.392897449732544 | 1.17056936822883e-05 | 0.032 | GO:0022610 | biological adhesion |
| 44 | 1099 | 0.389550344285613 | 6.52059983351516e-07 | 0.002 | GO:0080134 | regulation of response to stress |
| 283 | 10968 | 0.388466615437157 | 4.3684440983648e-18 | < 0.001 | GO:0043226 | organelle |
| 356 | 16725 | 0.387721964877707 | 1.66950146956973e-11 | < 0.001 | GO:0005575 | cellular component |
| 296 | 11863 | 0.386549428657223 | 3.01212289858378e-17 | < 0.001 | GO:0044424 | intracellular part |

*Continued on next page*

Supplementary Table 3 – *Continued from previous page*

| N | X | LOD | P | P adj | attrib ID | attrib name |
|---|---|---|---|---|---|---|
| 48 | 1213 | 0.386411067525816 | 2.71997402127956e-07 | $< 0.001$ | GO:0042325 | regulation of phosphorylation |
| 44 | 1114 | 0.383114524959309 | 9.36986284621682e-07 | 0.004 | GO:0001932 | regulation of protein phosphorylation |
| 48 | 1227 | 0.380937017161292 | 3.79461674808188e-07 | $< 0.001$ | GO:0051649 | establishment of localization in cell |
| 267 | 10119 | 0.376565153944642 | 1.2114372504688e-17 | $< 0.001$ | GO:0043227 | membrane-bounded organelle |
| 53 | 1379 | 0.375565775923956 | 1.53989006632191e-07 | $< 0.001$ | GO:0031090 | organelle membrane |
| 47 | 1227 | 0.370297203300555 | 9.15911813669104e-07 | 0.004 | GO:0042802 | identical protein binding |
| 75 | 2055 | 0.365047839485642 | 2.46950406297065e-09 | $< 0.001$ | GO:0006950 | response to stress |
| 40 | 1054 | 0.361950661490976 | 7.81821392787743e-06 | 0.025 | GO:0045184 | establishment of protein localization |
| 47 | 1252 | 0.360680509113436 | 1.59839643618343e-06 | 0.007 | GO:1902531 | regulation of intracellular signal transduction |
| 53 | 1424 | 0.360142447209269 | 4.19241955165934e-07 | $< 0.001$ | GO:0019220 | regulation of phosphate metabolic process |
| 58 | 1570 | 0.35965449613598 | 1.42601065414884e-07 | $< 0.001$ | GO:0048584 | positive regulation of response to stimulus |
| 53 | 1429 | 0.358458055659722 | 4.66942132985527e-07 | $< 0.001$ | GO:0051174 | regulation of phosphorus metabolic process |
| 50 | 1347 | 0.357055048558987 | 9.97549196945642e-07 | 0.004 | GO:0005102 | receptor binding |
| 213 | 7463 | 0.354133631925948 | 4.42310099188529e-16 | $< 0.001$ | GO:0005515 | protein binding |
| 43 | 1156 | 0.354067993554003 | 5.77296022298623e-06 | 0.015 | GO:0046983 | protein dimerization activity |
| 173 | 5722 | 0.345940186964955 | 1.8290681992227e-14 | $< 0.001$ | GO:0044422 | organelle part |
| 281 | 11392 | 0.345256112369023 | 1.19745449334275e-14 | $< 0.001$ | GO:0005488 | binding |
| 165 | 5512 | 0.332253321154314 | 3.07550977571961e-13 | $< 0.001$ | GO:0044446 | intracellular organelle part |
| 47 | 1331 | 0.331480694159866 | 8.18523240979299e-06 | 0.025 | GO:0033036 | macromolecule localization |
| 46 | 1318 | 0.325365080203981 | 1.38352732338407e-05 | 0.041 | GO:0008104 | protein localization |
| 208 | 7564 | 0.3234921375736 | 1.08539398735691e-13 | $< 0.001$ | GO:0016020 | membrane |
| 50 | 1447 | 0.322733475294741 | 7.43484613300299e-06 | 0.025 | GO:0031399 | regulation of protein modification process |
| 323 | 14779 | 0.309911772138204 | 3.62629630891498e-10 | $< 0.001$ | GO:0003674 | molecular function |
| 168 | 5914 | 0.303815334054725 | 1.65642757831703e-11 | $< 0.001$ | GO:0005737 | cytoplasm |
| 246 | 9827 | 0.302460890098254 | 3.040069437937e-12 | $< 0.001$ | GO:0043229 | intracellular organelle |
| 262 | 10790 | 0.300130623203149 | 7.25968090822923e-12 | $< 0.001$ | GO:0009987 | cellular process |
| 66 | 2101 | 0.285281853111959 | 5.61863755421225e-06 | 0.015 | GO:0009966 | regulation of signal transduction |
| 89 | 2936 | 0.28088041199165 | 4.10694733320769e-07 | $< 0.001$ | GO:0048583 | regulation of response to stimulus |
| 92 | 3116 | 0.26912758878425 | 7.95636138747502e-07 | 0.003 | GO:0051234 | establishment of localization |
| 88 | 2979 | 0.266998327993624 | 1.45904695286012e-06 | 0.007 | GO:0006810 | transport |
| 221 | 8912 | 0.265681513887642 | 6.77850244818075e-10 | $< 0.001$ | GO:0043231 | intracellular membrane-bounded organelle |
| 325 | 15496 | 0.260145200625659 | 1.9039857502456e-07 | $< 0.001$ | GO:0008150 | biological process |
| 99 | 3443 | 0.259557204168453 | 8.94747265642658e-07 | 0.003 | GO:0051179 | localization |
| 199 | 7864 | 0.258288559673377 | 2.5574950621635e-09 | $< 0.001$ | GO:0044763 | single-organism cellular process |
| 72 | 2444 | 0.256639550539202 | 1.80650749041464e-05 | 0.049 | GO:0023051 | regulation of signaling |
| 118 | 4271 | 0.249896554971121 | 4.27828192236721e-07 | $< 0.001$ | GO:0032991 | macromolecular complex |
| 106 | 3859 | 0.239418221465301 | 2.82869594808867e-06 | 0.012 | GO:0050896 | response to stimulus |

**Supplementary Table 4: GO categories enriched ($p < 0.05$) in genes upregulated in ASO II LPS treated relative to Control LPS cells**

| N | X | LOD | P | P adj | attrib ID | attrib name |
|---|---|---|---|---|---|---|
| 12 | 31 | 0.846043063523471 | 3.75533571153289e-06 | 0.009 | GO:0006335 | DNA replication-dependent nucleosome assembly |
| 12 | 31 | 0.846043063523471 | 3.75533571153289e-06 | 0.009 | GO:0034723 | DNA replication-dependent nucleosome organization |
| 11 | 30 | 0.809602795022447 | 1.75726744546686e-05 | 0.05 | GO:0031663 | lipopolysaccharide-mediated signaling pathway |
| 11 | 30 | 0.809602795022447 | 1.75726744546686e-05 | 0.05 | GO:0042116 | macrophage activation |
| 12 | 33 | 0.803597517029169 | 8.05310813391485e-06 | 0.022 | GO:0006487 | protein N-linked glycosylation |
| 13 | 40 | 0.730230136458929 | 1.39844434556007e-05 | 0.036 | GO:0006636 | unsaturated fatty acid biosynthetic process |
| 17 | 63 | 0.615331777061829 | 1.17677564472432e-05 | 0.031 | GO:0006664 | glycolipid metabolic process |
| 17 | 63 | 0.615331777061829 | 1.17677564472432e-05 | 0.031 | GO:0008652 | cellular amino acid biosynthetic process |
| 17 | 64 | 0.606070244799037 | 1.47772853433011e-05 | 0.04 | GO:0006497 | protein lipidation |
| 17 | 64 | 0.606070244799037 | 1.47772853433011e-05 | 0.04 | GO:1903509 | liposaccharide metabolic process |
| 20 | 76 | 0.599930106767911 | 3.13043617161542e-06 | 0.009 | GO:0046474 | glycerophospholipid biosynthetic process |
| 25 | 95 | 0.599430374658546 | 1.91694365845259e-07 | 0.001 | GO:0046488 | phosphatidylinositol metabolic process |
| 21 | 80 | 0.598312707155382 | 1.86874835682264e-06 | 0.005 | GO:0044815 | DNA packaging complex |
| 21 | 81 | 0.591053409650731 | 2.32432542233528e-06 | 0.007 | GO:0043021 | ribonucleoprotein complex binding |
| 30 | 120 | 0.569462532336769 | 4.23271002340507e-08 | < 0.001 | GO:0008654 | phospholipid biosynthetic process |
| 19 | 77 | 0.562832608396953 | 1.50633113682809e-05 | 0.04 | GO:0016051 | carbohydrate biosynthetic process |
| 25 | 103 | 0.552582609631034 | 1.00451230197177e-06 | 0.002 | GO:0006633 | fatty acid biosynthetic process |
| 21 | 87 | 0.549861786412747 | 7.90881260941229e-06 | 0.022 | GO:0045017 | glycerolipid biosynthetic process |
| 35 | 153 | 0.518888339004912 | 3.79332648719052e-08 | < 0.001 | GO:0006650 | glycerophospholipid metabolic process |
| 29 | 136 | 0.479638777870508 | 2.49994337214488e-06 | 0.007 | GO:0043123 | positive regulation of I-kappaB kinase/NF-kappaB signaling |
| 44 | 210 | 0.470400968980817 | 1.25154873715315e-08 | < 0.001 | GO:0016053 | organic acid biosynthetic process |
| 44 | 210 | 0.470400968980817 | 1.25154873715315e-08 | < 0.001 | GO:0046394 | carboxylic acid biosynthetic process |
| 37 | 178 | 0.465633242095294 | 2.16866165542015e-07 | 0.001 | GO:0005774 | vacuolar membrane |
| 47 | 228 | 0.461653849137166 | 6.98123471683876e-09 | < 0.001 | GO:0006644 | phospholipid metabolic process |
| 29 | 141 | 0.459790024991754 | 5.32726175932524e-06 | 0.012 | GO:0006260 | DNA replication |
| 72 | 353 | 0.458440200506168 | 1.26949262125656e-12 | < 0.001 | GO:0005773 | vacuole |
| 33 | 163 | 0.451099541515752 | 1.78398500068411e-06 | 0.004 | GO:0005765 | lysosomal membrane |
| 33 | 163 | 0.451099541515752 | 1.78398500068411e-06 | 0.004 | GO:0098852 | lytic vacuole membrane |
| 65 | 323 | 0.450200737786413 | 2.9003685360581e-11 | < 0.001 | GO:0044283 | small molecule biosynthetic process |
| 37 | 184 | 0.447471818697918 | 5.16631940868857e-07 | 0.001 | GO:0009116 | nucleoside metabolic process |
| 59 | 297 | 0.442464297442814 | 4.00276317180225e-10 | < 0.001 | GO:0090407 | organophosphate biosynthetic process |
| 33 | 166 | 0.441165903979251 | 2.71622162520181e-06 | 0.007 | GO:0010506 | regulation of autophagy |
| 29 | 147 | 0.437098530149385 | 1.24443982173983e-05 | 0.032 | GO:0061136 | regulation of proteasomal protein catabolic process |
| 38 | 194 | 0.433221379820495 | 7.26902752135876e-07 | 0.001 | GO:0044437 | vacuolar part |
| 38 | 195 | 0.430434177084543 | 8.30666781940227e-07 | 0.001 | GO:1901657 | glycosyl compound metabolic process |
| 49 | 255 | 0.423461517610518 | 3.63321420938e-08 | < 0.001 | GO:0006631 | fatty acid metabolic process |
| 33 | 172 | 0.421947167727636 | 6.05573721785567e-06 | 0.014 | GO:0009119 | ribonucleoside metabolic process |
| 59 | 310 | 0.419140701887449 | 2.22303620539841e-09 | < 0.001 | GO:0000323 | lytic vacuole |
| 59 | 310 | 0.419140701887449 | 2.22303620539841e-09 | < 0.001 | GO:0005764 | lysosome |
| 68 | 361 | 0.414468317773127 | 2.15083056451709e-10 | < 0.001 | GO:0008610 | lipid biosynthetic process |
| 43 | 229 | 0.41061349595705 | 4.72204798146345e-07 | 0.001 | GO:0098687 | chromosomal region |
| 151 | 834 | 0.403704516853496 | 7.05950913730947e-20 | < 0.001 | GO:0044711 | single-organism biosynthetic process |
| 34 | 186 | 0.395983902436579 | 1.30772601375915e-05 | 0.035 | GO:0043122 | regulation of I-kappaB kinase/NF-kappaB signaling |
| 51 | 283 | 0.389078951204161 | 1.63907072412149e-07 | 0.001 | GO:0051098 | regulation of binding |
| 45 | 252 | 0.383818914684149 | 1.1113582263383e-06 | 0.002 | GO:0006520 | cellular amino acid metabolic process |
| 44 | 247 | 0.382473240754282 | 1.55740698640442e-06 | 0.004 | GO:1901137 | carbohydrate derivative biosynthetic process |
| 38 | 215 | 0.378096434854134 | 9.42056370646506e-06 | 0.031 | GO:0051251 | positive regulation of lymphocyte activation |
| 46 | 261 | 0.376895205237804 | 1.23882855221221e-06 | 0.002 | GO:0007067 | mitotic nuclear division |
| 37 | 210 | 0.376418618698897 | 1.319587396024e-05 | 0.035 | GO:0046486 | glycerolipid metabolic process |
| 114 | 657 | 0.375628133701388 | 6.15648367379125e-14 | < 0.001 | GO:0019752 | carboxylic acid metabolic process |
| 47 | 271 | 0.368409703226569 | 1.52274795837918e-06 | 0.004 | GO:0016757 | transferase activity, transferring glycosyl groups |
| 82 | 476 | 0.367957356928879 | 3.1302030980019e-10 | < 0.001 | GO:1903047 | mitotic cell cycle process |
| 1215 | 10119 | 0.36697797274232 | 2.56695239725299e-68 | < 0.001 | GO:0043227 | membrane-bounded organelle |
| 54 | 314 | 0.36439262682441 | 3.45284715475785e-07 | 0.001 | GO:0000280 | nuclear division |
| 67 | 391 | 0.363584520356274 | 1.61738213514744e-08 | < 0.001 | GO:0005694 | chromosome |
| 1110 | 8912 | 0.362779830691097 | 9.00810946879018e-68 | < 0.001 | GO:0043231 | intracellular membrane-bounded organelle |
| 42 | 245 | 0.362038293844286 | 7.2351619791272e-06 | 0.02 | GO:0002696 | positive regulation of leukocyte activation |
| 44 | 257 | 0.361429386440298 | 4.58802896713741e-06 | 0.011 | GO:0050867 | positive regulation of cell activation |
| 62 | 367 | 0.355592927216533 | 9.33940471528317e-08 | 0.001 | GO:0044454 | nuclear chromosome part |
| 108 | 646 | 0.354929102906929 | 3.24774105844591e-12 | < 0.001 | GO:0044255 | cellular lipid metabolic process |
| 65 | 386 | 0.354149605472501 | 5.21051134989452e-08 | < 0.001 | GO:0019866 | organelle inner membrane |
| 46 | 273 | 0.353108453426715 | 4.37843264574343e-06 | 0.011 | GO:0045087 | innate immune response |
| 57 | 339 | 0.352689448137155 | 3.56848244116235e-07 | 0.001 | GO:0048285 | organelle fission |
| 1268 | 10968 | 0.352550920838068 | 4.690082597255e-62 | < 0.001 | GO:0043226 | organelle |
| 1176 | 9827 | 0.350904372417427 | 3.68124782750711e-63 | < 0.001 | GO:0043229 | intracellular organelle |
| 86 | 517 | 0.349747997164598 | 6.99136545589715e-10 | < 0.001 | GO:0098805 | whole membrane |
| 1329 | 11863 | 0.346338780257927 | 4.15441738090165e-58 | < 0.001 | GO:0044424 | intracellular part |
| 116 | 708 | 0.345093005441802 | 1.96885522867747e-12 | < 0.001 | GO:0043436 | oxoacid metabolic process |
| 96 | 584 | 0.344541782683312 | 1.37777786634181e-10 | < 0.001 | GO:0019637 | organophosphate metabolic process |
| 75 | 456 | 0.342623822016203 | 1.40987900850225e-08 | < 0.001 | GO:0031329 | regulation of cellular catabolic process |
| 67 | 408 | 0.341048682759899 | 8.76574119403513e-08 | 0.001 | GO:0044723 | single-organism carbohydrate metabolic process |
| 55 | 335 | 0.339975291241117 | 1.22965554560659e-06 | 0.002 | GO:0006281 | DNA repair |
| 117 | 722 | 0.339341783915065 | 3.31080862757364e-12 | < 0.001 | GO:0006082 | organic acid metabolic process |
| 64 | 392 | 0.337730888691993 | 2.09985723402641e-07 | 0.001 | GO:0032787 | monocarboxylic acid metabolic process |
| 59 | 362 | 0.336422305874606 | 6.56258082787453e-07 | 0.001 | GO:0005743 | mitochondrial inner membrane |
| 108 | 670 | 0.335478844739786 | 3.20318225435195e-11 | < 0.001 | GO:0044427 | chromosomal part |
| 63 | 389 | 0.333404909045799 | 3.48940777877058e-07 | 0.001 | GO:0051301 | cell division |

*Continued on next page*

| N | X | LOD | P | P adj | attrib ID | attrib name |
|---|---|---|---|---|---|---|
| 91 | 567 | 0.331301281139383 | 1.45994931343662e-09 | < 0.001 | GO:0006259 | DNA metabolic process |
| 215 | 1379 | 0.330899656528473 | 1.26326490388069e-19 | < 0.001 | GO:0031090 | organelle membrane |
| 59 | 370 | 0.324953828383564 | 1.36694408225549e-06 | 0.002 | GO:0004674 | protein serine/threonine kinase activity |
| 103 | 656 | 0.320987259412617 | 4.22282289130171e-10 | < 0.001 | GO:0007049 | cell cycle |
| 136 | 873 | 0.320560284218998 | 1.08681241587089e-12 | < 0.001 | GO:0006629 | lipid metabolic process |
| 1435 | 13700 | 0.320390140576835 | 1.0034221912607e-45 | < 0.001 | GO:0044464 | cell part |
| 48 | 305 | 0.317434058816055 | 1.81247593648814e-05 | 0.05 | GO:0042176 | regulation of protein catabolic process |
| 84 | 537 | 0.317049436354494 | 2.08284074349791e-08 | < 0.001 | GO:0006974 | cellular response to DNA damage stimulus |
| 96 | 620 | 0.3128923652287 | 3.47930821241571e-09 | < 0.001 | GO:0005768 | endosome |
| 130 | 846 | 0.312575311655455 | 9.28390663458269e-12 | < 0.001 | GO:0098588 | bounding membrane of organelle |
| 82 | 530 | 0.311093973401162 | 5.01005625396735e-08 | < 0.001 | GO:1901135 | carbohydrate derivative metabolic process |
| 196 | 1301 | 0.309869763153209 | 2.46500529214835e-16 | < 0.001 | GO:0044281 | small molecule metabolic process |
| 76 | 492 | 0.309710652919976 | 1.64182980496175e-07 | 0.001 | GO:0005975 | carbohydrate metabolic process |
| 69 | 450 | 0.305248184772817 | 7.93483831328104e-07 | 0.001 | GO:0010564 | regulation of cell cycle process |
| 720 | 5512 | 0.30522725919993 | 1.17730214082545e-42 | < 0.001 | GO:0044446 | intracellular organelle part |
| 144 | 954 | 0.304682223989538 | 2.63962242054648e-12 | < 0.001 | GO:0015031 | protein transport |
| 86 | 564 | 0.303846078005399 | 4.64351416987144e-08 | < 0.001 | GO:0009894 | regulation of catabolic process |
| 133 | 881 | 0.303509993285743 | 1.86672321533493e-11 | < 0.001 | GO:0016310 | phosphorylation |
| 112 | 739 | 0.30327896500303 | 6.11729699499123e-10 | < 0.001 | GO:0022402 | cell cycle process |
| 448 | 3207 | 0.301828515577143 | 1.76187459561221e-30 | < 0.001 | GO:0044710 | single-organism metabolic process |
| 219 | 1489 | 0.299729768793589 | 5.57307055481093e-17 | < 0.001 | GO:0006796 | phosphate-containing compound metabolic process |
| 99 | 657 | 0.298900055734507 | 8.33390816011829e-09 | < 0.001 | GO:1901566 | organonitrogen compound biosynthetic process |
| 66 | 436 | 0.298319652403335 | 2.14996317458061e-06 | 0.006 | GO:0051347 | positive regulation of transferase activity |
| 224 | 1531 | 0.297504988813604 | 4.29903624623456e-17 | < 0.001 | GO:0006793 | phosphorus metabolic process |
| 125 | 838 | 0.296280976445744 | 1.70096449634067e-10 | < 0.001 | GO:0016772 | transferase activity, transferring phosphorus-containing groups |
| 276 | 1916 | 0.295546148492557 | 4.50453854796639e-20 | < 0.001 | GO:0016740 | transferase activity |
| 64 | 426 | 0.294238151426327 | 3.93577651496503e-06 | 0.009 | GO:0006886 | intracellular protein transport |
| 213 | 1461 | 0.294233674617129 | 4.16905203967541e-16 | < 0.001 | GO:0071702 | organic substance transport |
| 73 | 488 | 0.292694082591889 | 9.95994902933601e-07 | 0.002 | GO:0031966 | mitochondrial membrane |
| 60 | 401 | 0.291858780805667 | 8.85336130230376e-06 | 0.028 | GO:0044432 | endoplasmic reticulum part |
| 154 | 1054 | 0.288515903547892 | 5.93720131383296e-12 | < 0.001 | GO:0045184 | establishment of protein localization |
| 727 | 5722 | 0.288434790254404 | 1.04340469221131e-38 | < 0.001 | GO:0044422 | organelle part |
| 189 | 1316 | 0.283310065497923 | 9.67123043348984e-14 | < 0.001 | GO:0005783 | endoplasmic reticulum |
| 81 | 554 | 0.281470811118308 | 6.6686285840238e-07 | 0.001 | GO:0006955 | immune response |
| 102 | 702 | 0.280129044475239 | 3.33061147607445e-08 | < 0.001 | GO:0006468 | protein phosphorylation |
| 160 | 1114 | 0.280074956450025 | 8.89310783445947e-12 | < 0.001 | GO:0002376 | immune system process |
| 73 | 500 | 0.280073893190563 | 2.47398606263057e-06 | 0.007 | GO:0010638 | positive regulation of organelle organization |
| 160 | 1120 | 0.277222534136814 | 1.36293439549773e-11 | < 0.001 | GO:0005794 | Golgi apparatus |
| 97 | 672 | 0.276217555191328 | 9.96316820383602e-08 | 0.001 | GO:0044429 | mitochondrial part |
| 900 | 7564 | 0.275911479511714 | 4.03496076456682e-39 | < 0.001 | GO:0016020 | membrane |
| 98 | 685 | 0.271668400761039 | 1.31751293933078e-07 | 0.001 | GO:0051338 | regulation of transferase activity |
| 105 | 740 | 0.268022863014269 | 7.15011923881232e-08 | < 0.001 | GO:0051726 | regulation of cell cycle |
| 397 | 2978 | 0.267776869091061 | 1.1668428816567e-22 | < 0.001 | GO:0044428 | nuclear part |
| 101 | 712 | 0.26751238775697 | 1.28745557221136e-07 | 0.001 | GO:0016301 | kinase activity |
| 790 | 6543 | 0.265529295782192 | 1.07496077704056e-34 | < 0.001 | GO:0044238 | primary metabolic process |
| 1589 | 16725 | 0.264330118461731 | 6.53641927035309e-25 | < 0.001 | GO:0005575 | cellular component |
| 74 | 523 | 0.263916953157834 | 6.66878545145911e-06 | 0.014 | GO:0044451 | nucleoplasm part |
| 814 | 6824 | 0.261444518929276 | 3.63643174425806e-34 | < 0.001 | GO:0071704 | organic substance metabolic process |
| 754 | 6248 | 0.259004096342968 | 1.87586291489573e-32 | < 0.001 | GO:0044237 | cellular metabolic process |
| 1163 | 10790 | 0.255874666962145 | 1.51924601556368e-34 | < 0.001 | GO:0009987 | cellular process |
| 756 | 6305 | 0.255018330275011 | 1.35097307571377e-31 | < 0.001 | GO:0044444 | cytoplasmic part |
| 220 | 1631 | 0.253115007527855 | 5.46421532055704e-13 | < 0.001 | GO:0005739 | mitochondrion |
| 130 | 951 | 0.250913197432285 | 1.91118667274497e-08 | < 0.001 | GO:0033043 | regulation of organelle organization |
| 91 | 663 | 0.249537562454216 | 2.23480742094585e-06 | 0.007 | GO:0016773 | phosphotransferase activity, alcohol group as acceptor |
| 178 | 1318 | 0.249315758823243 | 1.02762657048325e-10 | < 0.001 | GO:0008104 | protein localization |
| 852 | 7358 | 0.247294874135515 | 2.06476516739068e-31 | < 0.001 | GO:0008152 | metabolic process |
| 179 | 1331 | 0.247181888000703 | 1.27754378960775e-10 | < 0.001 | GO:0033036 | macromolecule localization |
| 267 | 2023 | 0.246494971368036 | 1.64166422870895e-14 | < 0.001 | GO:0043412 | macromolecule modification |
| 215 | 1620 | 0.243858902877784 | 5.07436709767787e-12 | < 0.001 | GO:0005654 | nucleoplasm |
| 137 | 1019 | 0.242879557212468 | 2.20346342510310e-08 | < 0.001 | GO:0033554 | cellular response to stress |
| 252 | 1919 | 0.242100581083728 | 1.81197892850054e-13 | < 0.001 | GO:0006464 | cellular protein modification process |
| 252 | 1919 | 0.242100581083728 | 1.81197892850054e-13 | < 0.001 | GO:0036211 | protein modification process |
| 157 | 1178 | 0.240211578130644 | 3.60910422453874e-09 | < 0.001 | GO:1901564 | organonitrogen compound metabolic process |
| 283 | 2188 | 0.237196991614965 | 2.89523804250904e-14 | < 0.001 | GO:0044267 | cellular protein metabolic process |
| 177 | 1347 | 0.234688372198871 | 1.01762687295828e-09 | < 0.001 | GO:0005524 | ATP binding |
| 86 | 647 | 0.232605277765759 | 1.46830388463405e-05 | 0.038 | GO:0002684 | positive regulation of immune system process |
| 393 | 3141 | 0.231030250241024 | 1.64108188544329e-17 | < 0.001 | GO:0009058 | biosynthetic process |
| 385 | 3079 | 0.229744994509276 | 4.35207233256838e-17 | < 0.001 | GO:1901576 | organic substance biosynthetic process |
| 181 | 1393 | 0.229128890973484 | 1.56098798356616e-09 | < 0.001 | GO:0030554 | adenyl nucleotide binding |
| 179 | 1385 | 0.226103318467121 | 2.91612875246828e-09 | < 0.001 | GO:0032559 | adenyl ribonucleotide binding |
| 585 | 4928 | 0.223353500617017 | 1.14898935139765e-21 | < 0.001 | GO:0003824 | catalytic activity |
| 371 | 2996 | 0.222638334463225 | 8.91172215557943e-16 | < 0.001 | GO:0044249 | cellular biosynthetic process |
| 873 | 7864 | 0.22036404648897 | 1.15199906941665e-25 | < 0.001 | GO:0044763 | single-organism cellular process |
| 141 | 1096 | 0.220204602794956 | 2.02045820205251e-07 | 0.001 | GO:0002682 | regulation of immune system process |
| 210 | 1653 | 0.219953183046986 | 4.85450227312016e-10 | < 0.001 | GO:0035639 | purine ribonucleoside triphosphate binding |
| 212 | 1670 | 0.219744163140906 | 4.24247684571767e-10 | < 0.001 | GO:0001883 | purine nucleoside binding |
| 211 | 1667 | 0.218081464931894 | 6.05436923622394e-10 | < 0.001 | GO:0032550 | purine ribonucleoside binding |
| 366 | 2979 | 0.217625198869584 | 4.86787232567061e-15 | < 0.001 | GO:0006810 | transport |
| 212 | 1677 | 0.217507980724696 | 6.11777625278648e-10 | < 0.001 | GO:0001882 | nucleoside binding |
| 211 | 1669 | 0.217440732804623 | 6.71878882910405e-10 | < 0.001 | GO:0032549 | ribonucleoside binding |
| 381 | 3116 | 0.21654125576573 | 2.33239636216498e-15 | < 0.001 | GO:0051234 | establishment of localization |

*Continued on next page*

Supplementary Table 4 – *Continued from previous page*

| N | X | LOD | P | P adj | attrib ID | attrib name |
|---|---|---|---|---|---|---|
| 359 | 2924 | 0.216484590924295 | 1.06610503205145e-14 | < 0.001 | GO:0019538 | protein metabolic process |
| 215 | 1713 | 0.213950967075064 | 8.56179733803621e-10 | < 0.001 | GO:0032553 | ribonucleotide binding |
| 118 | 926 | 0.213321319318208 | 3.24821454338026e-06 | 0.009 | GO:0031401 | positive regulation of protein modification process |
| 214 | 1709 | 0.212613320412264 | 1.15212264232723e-09 | < 0.001 | GO:0017076 | purine nucleotide binding |
| 152 | 1202 | 0.212174480092757 | 1.94398790599079e-07 | 0.001 | GO:0044093 | positive regulation of molecular function |
| 212 | 1699 | 0.210543681939746 | 1.87915328443066e-09 | < 0.001 | GO:0032555 | purine ribonucleotide binding |
| 118 | 931 | 0.210535429380526 | 4.20939491223875e-06 | 0.011 | GO:0043085 | positive regulation of catalytic activity |
| 157 | 1248 | 0.209890396307231 | 1.68314601775551e-07 | 0.001 | GO:0051247 | positive regulation of protein metabolic process |
| 145 | 1152 | 0.209140282925709 | 4.94347020926221e-07 | 0.001 | GO:0032270 | positive regulation of cellular protein metabolic process |
| 610 | 5288 | 0.208579318121639 | 1.03318200222905e-19 | < 0.001 | GO:0005634 | nucleus |
| 260 | 2114 | 0.207325296851094 | 9.05883849264766e-11 | < 0.001 | GO:0000166 | nucleotide binding |
| 260 | 2114 | 0.207325296851094 | 9.05883849264766e-11 | < 0.001 | GO:1901265 | nucleoside phosphate binding |
| 398 | 3344 | 0.20295537798911 | 3.15534737999326e-14 | < 0.001 | GO:0031982 | vesicle |
| 1467 | 15496 | 0.201653352606333 | 5.6545024473777e-18 | < 0.001 | GO:0008150 | biological process |
| 313 | 2599 | 0.200951558071282 | 9.14980594374575e-12 | < 0.001 | GO:1903561 | extracellular vesicle |
| 313 | 2601 | 0.200525056873849 | 1.00369348457354e-11 | < 0.001 | GO:0043230 | extracellular organelle |
| 407 | 3443 | 0.199982650610827 | 4.2000907046874e-14 | < 0.001 | GO:0051179 | localization |
| 311 | 2586 | 0.19998200229214 | 1.2810215324398e-11 | < 0.001 | GO:0070062 | extracellular exosome |
| 311 | 2588 | 0.199553694048285 | 1.40456262062794e-11 | < 0.001 | GO:0065010 | extracellular membrane-bounded organelle |
| 549 | 4782 | 0.198224184069335 | 6.0217798060244e-17 | < 0.001 | GO:0044260 | cellular macromolecule metabolic process |
| 291 | 2421 | 0.197754748870515 | 7.23131608043512e-11 | < 0.001 | GO:0036094 | small molecule binding |
| 247 | 2055 | 0.19365444344656 | 2.57476174024657e-09 | < 0.001 | GO:0006950 | response to stress |
| 356 | 3020 | 0.193207557456889 | 4.03267701436372e-12 | < 0.001 | GO:0031988 | membrane-bounded vesicle |
| 202 | 1682 | 0.189273338126471 | 9.28601764949395e-08 | 0.001 | GO:0019899 | enzyme binding |
| 603 | 5394 | 0.187838489604475 | 2.46272650723403e-16 | < 0.001 | GO:0043170 | macromolecule metabolic process |
| 151 | 1252 | 0.187362573681841 | 3.41888295482421e-06 | 0.009 | GO:1902531 | regulation of intracellular signal transduction |
| 456 | 3998 | 0.184186256034251 | 2.72535738290315e-13 | < 0.001 | GO:0006807 | nitrogen compound metabolic process |
| 417 | 3636 | 0.183500564587319 | 1.92933164015578e-12 | < 0.001 | GO:0043234 | protein complex |
| 425 | 3714 | 0.182999301352442 | 1.5165963359881e-12 | < 0.001 | GO:0034641 | cellular nitrogen compound metabolic process |
| 242 | 2067 | 0.179040268035388 | 3.8698673340503e-08 | < 0.001 | GO:0006996 | organelle organization |
| 183 | 1558 | 0.175990846830916 | 1.69845881419329e-06 | 0.004 | GO:0050790 | regulation of catalytic activity |
| 381 | 3358 | 0.173885484019957 | 1.01903561406902e-10 | < 0.001 | GO:0046483 | heterocycle metabolic process |
| 405 | 3584 | 0.173784714664005 | 3.67288959003166e-11 | < 0.001 | GO:1901360 | organic cyclic compound metabolic process |
| 257 | 2226 | 0.172742265296542 | 4.76575157237787e-08 | < 0.001 | GO:0044765 | single-organism transport |
| 383 | 3395 | 0.170796162224864 | 1.88379840421789e-10 | < 0.001 | GO:0006725 | cellular aromatic compound metabolic process |
| 234 | 2037 | 0.168167810991115 | 3.1008973072744e-07 | 0.001 | GO:0097367 | carbohydrate derivative binding |
| 363 | 3223 | 0.168074087863977 | 8.02633420339376e-10 | < 0.001 | GO:0006139 | nucleobase-containing compound metabolic process |
| 300 | 2638 | 0.168065211269837 | 1.31420283554086e-08 | < 0.001 | GO:0043228 | non-membrane-bounded organelle |
| 300 | 2638 | 0.168065211269837 | 1.31420283554086e-08 | < 0.001 | GO:0043232 | intracellular non-membrane-bounded organelle |
| 167 | 1447 | 0.165571513363832 | 1.35527734409103e-05 | 0.035 | GO:0031399 | regulation of protein modification process |
| 635 | 5914 | 0.165473627968873 | 1.56252413516134e-13 | < 0.001 | GO:0005737 | cytoplasm |
| 233 | 2042 | 0.164474381638895 | 5.56372724558811e-07 | 0.001 | GO:0065009 | regulation of molecular function |
| 470 | 4271 | 0.164298184167036 | 3.15662395095055e-11 | < 0.001 | GO:0032991 | macromolecular complex |
| 273 | 2409 | 0.163927283735032 | 9.28268234706421e-08 | 0.001 | GO:1902578 | single-organism localization |
| 223 | 1958 | 0.162709894353811 | 1.15121190378763e-06 | 0.002 | GO:0051716 | cellular response to stimulus |
| 269 | 2398 | 0.158060032310356 | 2.84822809795146e-07 | 0.001 | GO:0043168 | anion binding |
| 1115 | 11392 | 0.156439522535707 | 3.86360009251934e-14 | < 0.001 | GO:0005488 | binding |
| 247 | 2215 | 0.153173066521733 | 1.49943935479482e-06 | 0.004 | GO:0051246 | regulation of protein metabolic process |
| 228 | 2045 | 0.15168665981018 | 4.05496567600769e-06 | 0.009 | GO:0032268 | regulation of cellular protein metabolic process |
| 320 | 2936 | 0.146104345469586 | 2.86151143035925e-07 | 0.001 | GO:0048583 | regulation of response to stimulus |
| 231 | 2101 | 0.14442991628834 | 9.267588735101193e-06 | 0.03 | GO:0009966 | regulation of signal transduction |
| 508 | 4800 | 0.144153242301266 | 1.58594195582134e-09 | < 0.001 | GO:0048518 | positive regulation of biological process |
| 274 | 2511 | 0.143366461350823 | 2.21677445959537e-06 | 0.007 | GO:0010604 | positive regulation of macromolecule metabolic process |
| 310 | 2857 | 0.142871245506145 | 6.9181821219095e-07 | 0.001 | GO:0090304 | nucleic acid metabolic process |
| 271 | 2490 | 0.141728627583011 | 3.13248445616154e-06 | 0.009 | GO:0010646 | regulation of cell communication |
| 226 | 2068 | 0.14085036084975 | 1.7534671021737e-05 | 0.049 | GO:0051128 | regulation of cellular component organization |
| 265 | 2437 | 0.140831208370375 | 4.38988635454959e-06 | 0.011 | GO:0044271 | cellular nitrogen compound biosynthetic process |
| 515 | 4905 | 0.13987459806683 | 3.75992626746367e-09 | < 0.001 | GO:1901363 | heterocyclic compound binding |
| 318 | 2950 | 0.139812806937621 | 8.80124115127862e-07 | 0.001 | GO:0009893 | positive regulation of metabolic process |
| 1366 | 14779 | 0.139526804407888 | 2.71874376849952e-10 | < 0.001 | GO:0003674 | molecular function |
| 520 | 4974 | 0.13764308310875 | 5.71108264188005e-09 | < 0.001 | GO:0097159 | organic cyclic compound binding |
| 252 | 2339 | 0.134925944669782 | 1.52310925073869e-05 | 0.04 | GO:0009059 | macromolecule biosynthetic process |
| 450 | 4289 | 0.134176910113064 | 6.34819334023839e-08 | < 0.001 | GO:0048522 | positive regulation of cellular process |
| 271 | 2536 | 0.131746138870997 | 1.28389894080423e-05 | 0.034 | GO:0031325 | positive regulation of cellular metabolic process |
| 1042 | 10882 | 0.125603029899555 | 8.43004671690729e-10 | < 0.001 | GO:0044699 | single-organism process |
| 743 | 7463 | 0.124924065434313 | 4.10116301417074e-09 | < 0.001 | GO:0005515 | protein binding |
| 402 | 3898 | 0.120562032576895 | 2.59803256744903e-06 | 0.007 | GO:0071840 | cellular component organization or biogenesis |
| 394 | 3836 | 0.117662453752563 | 5.06720366272364e-06 | 0.012 | GO:0016043 | cellular component organization |
| 502 | 5062 | 0.103762207402661 | 9.6220751327613e-06 | 0.031 | GO:0043167 | ion binding |
| 525 | 5331 | 0.100898968097438 | 1.2105242297713e-05 | 0.032 | GO:0019222 | regulation of metabolic process |

**Supplementary Table 5: GO categories enriched ($p < 0.05$) in genes downregulated in ASO II LPS treated relative to Control LPS cells**

| N | X | LOD | P | P adj | attrib ID | attrib name |
|---|---|---|---|---|---|---|
| 8 | 12 | 1.32656238014753 | 7.4197390230586e-07 | 0.004 | GO:0070034 | telomerase RNA binding |
| 10 | 17 | 1.19688852879016 | 1.53645568262726e-07 | 0.002 | GO:0022624 | proteasome accessory complex |
| 11 | 21 | 1.09044030975503 | 1.79541208798984e-07 | 0.002 | GO:0097525 | spliceosomal snRNP complex |
| 13 | 26 | 1.05133702948679 | 2.75762574476408e-08 | 0.001 | GO:0030532 | small nuclear ribonucleoprotein complex |
| 33 | 70 | 1.00655546032383 | 5.54268050333076e-18 | < 0.001 | GO:0071013 | catalytic step 2 spliceosome |
| 10 | 22 | 0.974935871028536 | 3.48068083981087e-06 | 0.013 | GO:0005689 | U12-type spliceosomal complex |
| 56 | 135 | 0.911821753861585 | 4.8176394175761e-26 | < 0.001 | GO:0005681 | spliceosomal complex |
| 24 | 59 | 0.892398151503505 | 9.66894773130053e-12 | < 0.001 | GO:0000375 | RNA splicing, via transesterification reactions |
| 28 | 69 | 0.891074649776634 | 1.96143151779829e-13 | < 0.001 | GO:0042254 | ribosome biogenesis |
| 23 | 58 | 0.874064666955388 | 4.8685106158874e-11 | < 0.001 | GO:0000377 | RNA splicing, via transesterification reactions with bulged adenosine as nucleophile |
| 23 | 58 | 0.874064666955388 | 4.8685106158874e-11 | < 0.001 | GO:0000398 | mRNA splicing, via spliceosome |
| 17 | 43 | 0.871795650623963 | 1.71916827383893e-08 | < 0.001 | GO:0022625 | cytosolic large ribosomal subunit |
| 32 | 86 | 0.83043846728793 | 6.85509131384605e-14 | < 0.001 | GO:0022613 | ribonucleoprotein complex biogenesis |
| 18 | 49 | 0.820995148051562 | 2.56010095218152e-08 | < 0.001 | GO:0006352 | DNA-templated transcription, initiation |
| 87 | 241 | 0.819141380099197 | 2.99232218039153e-34 | < 0.001 | GO:0008380 | RNA splicing |
| 21 | 58 | 0.811120590974776 | 2.41388394710016e-09 | < 0.001 | GO:0000502 | proteasome complex |
| 13 | 36 | 0.810395034855466 | 2.805147181188e-06 | 0.013 | GO:0005747 | mitochondrial respiratory chain complex I |
| 13 | 36 | 0.810395034855466 | 2.805147181188e-06 | 0.013 | GO:0030964 | NADH dehydrogenase complex |
| 13 | 36 | 0.810395034855466 | 2.805147181188e-06 | 0.013 | GO:0045271 | respiratory chain complex I |
| 12 | 34 | 0.79564354922307 | 8.91971058812514e-06 | 0.034 | GO:0003743 | translation initiation factor activity |
| 15 | 43 | 0.78698009493078 | 8.09304190355766e-07 | 0.006 | GO:0044452 | nucleolar part |
| 21 | 62 | 0.767020517965535 | 9.6508645607201e-09 | < 0.001 | GO:0070469 | respiratory chain |
| 16 | 48 | 0.757244879163553 | 7.08491230210858e-07 | 0.004 | GO:0006413 | translational initiation |
| 21 | 63 | 0.756658871127748 | 1.33612942708553e-08 | < 0.001 | GO:0051082 | unfolded protein binding |
| 18 | 55 | 0.745149597985848 | 1.9800755729835e-07 | 0.003 | GO:0008135 | translation factor activity, RNA binding |
| 35 | 110 | 0.727505075831631 | 1.00577202196301e-12 | < 0.001 | GO:0016072 | rRNA metabolic process |
| 33 | 104 | 0.725572602923653 | 4.84337682106755e-12 | < 0.001 | GO:0006364 | rRNA processing |
| 318 | 1112 | 0.715751405040075 | 4.32359869149365e-95 | < 0.001 | GO:0044822 | poly(A) RNA binding |
| 20 | 64 | 0.715726680603163 | 1.00318090209295e-07 | 0.001 | GO:0070603 | SWI/SNF superfamily-type complex |
| 95 | 316 | 0.7012406548667 | 5.08998226018482e-30 | < 0.001 | GO:0006397 | mRNA processing |
| 36 | 119 | 0.695900057391436 | 2.58443528991364e-12 | < 0.001 | GO:0044085 | cellular component biogenesis |
| 15 | 50 | 0.691450977176312 | 6.93616814950691e-06 | 0.025 | GO:0098803 | respiratory chain complex |
| 19 | 64 | 0.684100474961578 | 5.11108491247865e-07 | 0.004 | GO:0017048 | Rho GTPase binding |
| 20 | 69 | 0.669377400209132 | 3.96623127150515e-07 | 0.004 | GO:0015934 | large ribosomal subunit |
| 380 | 1472 | 0.66589774317532 | 7.23912110182384e-100 | < 0.001 | GO:0003723 | RNA binding |
| 163 | 588 | 0.663894863492474 | 7.69033018196041e-46 | < 0.001 | GO:0030529 | intracellular ribonucleoprotein complex |
| 163 | 588 | 0.663894863492474 | 7.69033018196041e-46 | < 0.001 | GO:1990904 | ribonucleoprotein complex |
| 35 | 123 | 0.658237661571979 | 3.5704574755848e-11 | < 0.001 | GO:0006457 | protein folding |
| 17 | 60 | 0.656198556754772 | 4.10907048280778e-06 | 0.02 | GO:0015935 | small ribosomal subunit |
| 36 | 128 | 0.651257064766265 | 2.72554863585965e-11 | < 0.001 | GO:0044391 | ribosomal subunit |
| 104 | 386 | 0.635634601837924 | 2.32997748696915e-28 | < 0.001 | GO:0016071 | mRNA metabolic process |
| 21 | 77 | 0.63270785958031 | 6.30556822582225e-07 | 0.004 | GO:0007030 | Golgi organization |
| 29 | 107 | 0.628695382537329 | 5.75595142141495e-09 | < 0.001 | GO:0022618 | ribonucleoprotein complex assembly |
| 31 | 115 | 0.625543937871822 | 2.00494958225724e-09 | < 0.001 | GO:0071826 | ribonucleoprotein complex subunit organization |
| 26 | 98 | 0.616068652325708 | 5.6028745094722e-08 | 0.001 | GO:0006338 | chromatin remodeling |
| 44 | 167 | 0.613050797510759 | 2.09489795842475e-12 | < 0.001 | GO:0005840 | ribosome |
| 137 | 535 | 0.611156202005857 | 1.63216371394809e-34 | < 0.001 | GO:0006396 | RNA processing |
| 21 | 80 | 0.610176853256429 | 1.2539632099566e-06 | 0.008 | GO:0043130 | ubiquitin binding |
| 24 | 94 | 0.593708530677519 | 3.90501875090008e-07 | 0.004 | GO:0016607 | nuclear speck |
| 19 | 75 | 0.589834376505218 | 7.01153018868339e-06 | 0.027 | GO:0031397 | negative regulation of protein ubiquitination |
| 24 | 95 | 0.587570763400976 | 4.8218880845367e-07 | 0.004 | GO:0010970 | microtubule-based transport |
| 24 | 95 | 0.587570763400976 | 4.8218880845367e-07 | 0.004 | GO:0030705 | cytoskeleton-dependent intracellular transport |
| 19 | 76 | 0.582194151162435 | 8.63522469714114e-06 | 0.034 | GO:0016363 | nuclear matrix |
| 20 | 81 | 0.574857559668607 | 6.19086592725515e-06 | 0.024 | GO:1903321 | negative regulation of protein modification by small protein conjugation or removal |
| 20 | 82 | 0.567831824631562 | 7.56189685560012e-06 | 0.032 | GO:0003735 | structural constituent of ribosome |
| 31 | 128 | 0.563124818726945 | 3.17567911423402e-08 | 0.001 | GO:1903900 | regulation of viral life cycle |
| 152 | 652 | 0.558758821307337 | 3.61116917950793e-33 | < 0.001 | GO:0005730 | nucleolus |
| 37 | 159 | 0.540611671735892 | 4.95565169711632e-09 | < 0.001 | GO:0044445 | cytosolic part |
| 32 | 138 | 0.538401058594998 | 5.78373075087911e-08 | 0.001 | GO:0050792 | regulation of viral process |
| 35 | 151 | 0.538270534180428 | 1.42356907421444e-08 | < 0.001 | GO:0042393 | histone binding |
| 22 | 95 | 0.538093814394755 | 6.70030909869267e-06 | 0.024 | GO:0032182 | ubiquitin-like protein binding |
| 1439 | 11863 | 0.526689579279216 | 1.79640481493328e-118 | < 0.001 | GO:0044424 | intracellular part |
| 49 | 220 | 0.51688584734178 | 8.91001886119028e-11 | < 0.001 | GO:0016604 | nuclear body |
| 22 | 99 | 0.514996063096343 | 1.35449704352496e-05 | 0.047 | GO:0030496 | midbody |
| 59 | 269 | 0.509384227668778 | 2.17841827363148e-12 | < 0.001 | GO:0006511 | ubiquitin-dependent protein catabolic process |
| 555 | 2978 | 0.508288156312507 | 5.91795772242683e-88 | < 0.001 | GO:0044428 | nuclear part |
| 60 | 275 | 0.506562476582386 | 1.79869445375444e-12 | < 0.001 | GO:0019941 | modification-dependent protein catabolic process |
| 28 | 129 | 0.501406125355335 | 1.58157223619215e-06 | 0.008 | GO:0016050 | vesicle organization |
| 60 | 278 | 0.500495341165669 | 2.92739085534381e-12 | < 0.001 | GO:0043632 | modification-dependent macromolecule catabolic process |
| 67 | 317 | 0.489698530664776 | 4.46239221595389e-13 | < 0.001 | GO:0051603 | proteolysis involved in cellular protein catabolic process |
| 29 | 137 | 0.487509351077011 | 1.7821949197711e-06 | 0.009 | GO:0000209 | protein polyubiquitination |
| 108 | 523 | 0.482571006305815 | 1.71082942102201e-19 | < 0.001 | GO:0044451 | nucleoplasm part |
| 92 | 444 | 0.482101047203044 | 6.99972904795631e-17 | < 0.001 | GO:0016568 | chromatin modification |

*Continued on next page*

| N | X | LOD | P | P adj | attrib ID | attrib name |
|---|---|---|---|---|---|---|
| 42 | 202 | 0.47802228093892 | 1.65717238408239e-08 | < 0.001 | GO:1903320 | regulation of protein modification by small protein conjugation or removal |
| 38 | 183 | 0.477070458645462 | 8.12750490882431e-08 | 0.001 | GO:0031396 | regulation of protein ubiquitination |
| 1262 | 9827 | 0.476335520990951 | 6.41715382994507e-109 | < 0.001 | GO:0043229 | intracellular organelle |
| 29 | 140 | 0.47560157722655 | 2.82361405790647e-06 | 0.013 | GO:0003729 | mRNA binding |
| 860 | 5512 | 0.473958502004546 | 4.01886263134 25e-103 | < 0.001 | GO:0044446 | intracellular organelle part |
| 1336 | 10968 | 0.467231330948805 | 5.78865495028459e-101 | < 0.001 | GO:0043226 | organelle |
| 877 | 5722 | 0.466991152471787 | 2.81903084771396e-101 | < 0.001 | GO:0044422 | organelle part |
| 39 | 192 | 0.465031257778006 | 1.02405735203094e-07 | 0.001 | GO:0051656 | establishment of organelle localization |
| 33 | 166 | 0.453103188651204 | 1.58372305322541e-06 | 0.008 | GO:0016197 | endosomal transport |
| 48 | 243 | 0.450398322719836 | 9.29989267097238e-09 | < 0.001 | GO:0006412 | translation |
| 538 | 3165 | 0.44928988234603 | 1.63468766253073e-69 | < 0.001 | GO:0003676 | nucleic acid binding |
| 30 | 153 | 0.445672670995214 | 6.1934361273539e-06 | 0.024 | GO:0007018 | microtubule-based movement |
| 33 | 169 | 0.443389082932277 | 2.3945984179195e-06 | 0.013 | GO:0043903 | regulation of symbiosis, encompassing mutualism through parasitism |
| 295 | 1620 | 0.442903223390244 | 1.70342259488413e-41 | < 0.001 | GO:0005654 | nucleoplasm |
| 1254 | 10119 | 0.442256029228942 | 2.50063695377847e-94 | < 0.001 | GO:0043227 | membrane-bounded organelle |
| 92 | 478 | 0.441391727035042 | 8.42362648387663e-15 | < 0.001 | GO:0016482 | cytoplasmic transport |
| 80 | 415 | 0.440658724133064 | 4.44358247163047e-13 | < 0.001 | GO:0044265 | cellular macromolecule catabolic process |
| 36 | 186 | 0.438650496825279 | 1.06007552024739e-06 | 0.007 | GO:1903362 | regulation of cellular protein catabolic process |
| 801 | 5288 | 0.438510915743692 | 1.97101423258269e-86 | < 0.001 | GO:0005634 | nucleus |
| 1154 | 8912 | 0.436915774103139 | 1.89144121363023e-94 | < 0.001 | GO:0043231 | intracellular membrane-bounded organelle |
| 1483 | 13700 | 0.436200468603147 | 6.45522977640614e-76 | < 0.001 | GO:0044464 | cell part |
| 98 | 515 | 0.435991149998646 | 2.35938346846318e-15 | < 0.001 | GO:0006325 | chromatin organization |
| 50 | 261 | 0.433925854299454 | 1.32366448944792e-08 | < 0.001 | GO:0043043 | peptide biosynthetic process |
| 58 | 304 | 0.432462451682527 | 1.09982241680317e-09 | < 0.001 | GO:0033365 | protein localization to organelle |
| 65 | 343 | 0.429547916132737 | 1.48233020183478e-10 | < 0.001 | GO:0005925 | focal adhesion |
| 44 | 233 | 0.425727269088606 | 1.48034326784e-07 | 0.002 | GO:0044389 | ubiquitin-like protein ligase binding |
| 40 | 213 | 0.422516697341163 | 6.22335838958188e-07 | 0.004 | GO:0034470 | ncRNA processing |
| 66 | 353 | 0.422366841346918 | 1.94710317318841e-10 | < 0.001 | GO:0030055 | cell-substrate junction |
| 65 | 348 | 0.4217147791106 | 2.7879751362468e-10 | < 0.001 | GO:0005924 | cell-substrate adherens junction |
| 92 | 504 | 0.412566688107085 | 2.21161525345056e-13 | < 0.001 | GO:0009057 | macromolecule catabolic process |
| 654 | 4271 | 0.410553700131923 | 4.05747683417016e-68 | < 0.001 | GO:0032991 | macromolecular complex |
| 86 | 474 | 0.408518744740141 | 1.90736581895652e-12 | < 0.001 | GO:0070647 | protein modification by small protein conjugation or removal |
| 73 | 402 | 0.407418413769524 | 8.70167196555157e-11 | < 0.001 | GO:0005912 | adherens junction |
| 48 | 264 | 0.405646637684567 | 1.36880187754378e-07 | 0.001 | GO:0051640 | organelle localization |
| 135 | 760 | 0.403659161652798 | 4.72087217436947e-18 | < 0.001 | GO:1902494 | catalytic complex |
| 35 | 194 | 0.400936654701409 | 7.74102717930708e-06 | 0.032 | GO:0005096 | GTPase activator activity |
| 426 | 2638 | 0.397356052509461 | 5.63790119834636e-47 | < 0.001 | GO:0043228 | non-membrane-bounded organelle |
| 426 | 2638 | 0.397356052509461 | 5.63790119834636e-47 | < 0.001 | GO:0043232 | intracellular non-membrane-bounded organelle |
| 41 | 229 | 0.397021048363766 | 1.64394121924856e-06 | 0.009 | GO:0031625 | ubiquitin protein ligase binding |
| 50 | 281 | 0.394265348592654 | 1.52101205698605e-07 | 0.002 | GO:0016569 | covalent chromatin modification |
| 74 | 418 | 0.393878672527336 | 2.17368355663303e-10 | < 0.001 | GO:0070161 | anchoring junction |
| 36 | 203 | 0.391816360686853 | 8.61700201014521e-06 | 0.033 | GO:0003713 | transcription coactivator activity |
| 66 | 374 | 0.391307009392486 | 2.33778176711947e-09 | < 0.001 | GO:0032446 | protein modification by small protein conjugation |
| 41 | 232 | 0.390100738055517 | 2.31544769246397e-06 | 0.011 | GO:0033044 | regulation of chromosome organization |
| 35 | 198 | 0.390095851556116 | 1.22769783509578e-05 | 0.041 | GO:0032535 | regulation of cellular component size |
| 49 | 278 | 0.389150858019301 | 2.7127184790585e-07 | 0.003 | GO:0016570 | histone modification |
| 706 | 4905 | 0.383085655967195 | 4.07622008062035e-63 | < 0.001 | GO:1901363 | heterocyclic compound binding |
| 125 | 730 | 0.382408123716063 | 1.56631380122995e-15 | < 0.001 | GO:1902582 | single-organism intracellular transport |
| 57 | 330 | 0.379026448614194 | 6.13466858049715e-08 | 0.001 | GO:0006518 | peptide metabolic process |
| 147 | 870 | 0.378212250040635 | 1.46398921193336e-17 | < 0.001 | GO:0046907 | intracellular transport |
| 43 | 249 | 0.377951339345646 | 2.48314576248562e-06 | 0.013 | GO:0015631 | tubulin binding |
| 39 | 226 | 0.377426321371118 | 7.271423395757e-06 | 0.029 | GO:0031974 | membrane-enclosed lumen |
| 708 | 4974 | 0.376441359810842 | 2.91940792557695e-61 | < 0.001 | GO:0097159 | organic cyclic compound binding |
| 59 | 346 | 0.372379779273714 | 5.78859630459 74e-08 | 0.001 | GO:0016567 | protein ubiquitination |
| 82 | 486 | 0.369117374693671 | 2.68212764592483e-10 | < 0.001 | GO:1990234 | transferase complex |
| 1283 | 11392 | 0.368761612576796 | 6.27735548077474e-65 | < 0.001 | GO:0005488 | binding |
| 53 | 314 | 0.366493127138625 | 3.74619839621323e-07 | 0.004 | GO:0043604 | amide biosynthetic process |
| 81 | 485 | 0.363578140871852 | 5.69298610788388e-10 | < 0.001 | GO:0061024 | membrane organization |
| 47 | 280 | 0.36317420294078 | 1.96522000759493e-06 | 0.009 | GO:0010639 | negative regulation of organelle organization |
| 127 | 774 | 0.359747784166333 | 2.49186026667998e-14 | < 0.001 | GO:0051641 | cellular localization |
| 795 | 5914 | 0.358547088268804 | 1.16653533237034e-59 | < 0.001 | GO:0005737 | cytoplasm |
| 668 | 4782 | 0.356201788406312 | 1.41710298257026e-53 | < 0.001 | GO:0044260 | cellular macromolecule metabolic process |
| 87 | 530 | 0.355086028518224 | 3.21204185892518e-10 | < 0.001 | GO:0048471 | perinuclear region of cytoplasm |
| 60 | 365 | 0.35310549544194 | 1.69428061613525e-07 | 0.002 | GO:0003779 | actin binding |
| 88 | 539 | 0.352331019582281 | 3.36480143694664e-10 | < 0.001 | GO:0017111 | nucleoside-triphosphatase activity |
| 94 | 578 | 0.350947520186493 | 1.03625672064422e-10 | < 0.001 | GO:0016462 | pyrophosphatase activity |
| 94 | 578 | 0.350947520186493 | 1.03625672064422e-10 | < 0.001 | GO:0016818 | hydrolase activity, acting on acid anhydrides, in phosphorus-containing anhydrides |
| 1223 | 10790 | 0.350869176496287 | 1.57529046507314e-60 | < 0.001 | GO:0009987 | cellular process |
| 94 | 579 | 0.35003080235927 | 1.1400269636252e-10 | < 0.001 | GO:0016817 | hydrolase activity, acting on acid anhydrides |
| 58 | 355 | 0.349719085312101 | 3.33889445764393e-07 | 0.004 | GO:0008047 | enzyme activator activity |
| 369 | 2455 | 0.346614285965847 | 3.51233412219061e-33 | < 0.001 | GO:0016070 | RNA metabolic process |
| 315 | 2067 | 0.346044103055027 | 4.29932979954847e-29 | < 0.001 | GO:0006996 | organelle organization |
| 69 | 427 | 0.344903778186812 | 4.12432491789088e-08 | 0.001 | GO:0007017 | microtubule-based process |
| 48 | 298 | 0.341582594249546 | 4.99326312669178e-06 | 0.02 | GO:0070925 | organelle assembly |
| 88 | 551 | 0.340684199246932 | 1.04182736009826e-09 | < 0.001 | GO:0034613 | cellular protein localization |

*Continued on next page*

| N | X | LOD | P | P adj | attrib ID | attrib name |
|---|---|---|---|---|---|---|
| 47 | 293 | 0.339370996605756 | 6.98288844647657e-06 | 0.027 | GO:0034660 | ncRNA metabolic process |
| 418 | 2857 | 0.339009223460149 | 2.98379442881619e-35 | < 0.001 | GO:0090304 | nucleic acid metabolic process |
| 88 | 556 | 0.335918116477805 | 1.64385698987975e-09 | < 0.001 | GO:0070727 | cellular macromolecule localization |
| 62 | 391 | 0.334360007603504 | 3.90041365082454e-07 | 0.004 | GO:0005694 | chromosome |
| 48 | 303 | 0.332894289388028 | 7.92415970558983e-06 | 0.033 | GO:0032990 | cell part morphogenesis |
| 56 | 354 | 0.332654008075374 | 1.51238928233752e-06 | 0.008 | GO:0003712 | transcription cofactor activity |
| 53 | 335 | 0.332504413112462 | 2.84615679776584e-06 | 0.013 | GO:0006281 | DNA repair |
| 710 | 5394 | 0.326774639128547 | 1.58295096329463e-47 | < 0.001 | GO:0043170 | macromolecule metabolic process |
| 67 | 431 | 0.324405403183891 | 2.84781732478772e-07 | 0.003 | GO:0044802 | single-organism membrane organization |
| 515 | 3714 | 0.323549321542185 | 5.4747180556531e-38 | < 0.001 | GO:0034641 | cellular nitrogen compound metabolic process |
| 243 | 1630 | 0.323502843672629 | 5.63080007993451e-21 | < 0.001 | GO:0043933 | macromolecular complex subunit organization |
| 83 | 537 | 0.322917202499492 | 1.45071667591554e-08 | < 0.001 | GO:0006974 | cellular response to DNA damage stimulus |
| 536 | 3898 | 0.32208016222894 | 9.20020416408918e-39 | < 0.001 | GO:0071840 | cellular component organization or biogenesis |
| 118 | 775 | 0.31884820226946 | 3.3396105121369e-11 | < 0.001 | GO:0008092 | cytoskeletal protein binding |
| 59 | 384 | 0.317665567754866 | 2.10654522575529e-06 | 0.009 | GO:0051493 | regulation of cytoskeleton organization |
| 238 | 1612 | 0.317536627939688 | 5.36645971242373e-20 | < 0.001 | GO:1902589 | single-organism organelle organization |
| 450 | 3223 | 0.31703681405861 | 3.20021810717368e-33 | < 0.001 | GO:0006139 | nucleobase-containing compound metabolic process |
| 183 | 1227 | 0.315797053457893 | 6.51531276765809e-16 | < 0.001 | GO:0051649 | establishment of localization in cell |
| 499 | 3636 | 0.314772365709277 | 2.14284527705769e-35 | < 0.001 | GO:0043234 | protein complex |
| 63 | 415 | 0.31170468325375 | 1.46073536944084e-06 | 0.008 | GO:0034622 | cellular macromolecular complex assembly |
| 72 | 476 | 0.310578584132937 | 3.06293410516649e-07 | 0.004 | GO:1903047 | mitotic cell cycle process |
| 77 | 511 | 0.309044850203188 | 1.40527012040407e-07 | 0.001 | GO:1902580 | single-organism cellular localization |
| 75 | 498 | 0.308575442148337 | 2.08266260232601e-07 | 0.003 | GO:0005815 | microtubule organizing center |
| 77 | 513 | 0.307007955838482 | 1.65247082953229e-07 | 0.002 | GO:0008134 | transcription factor binding |
| 66 | 439 | 0.306899025063912 | 1.17167539826058e-06 | 0.008 | GO:0043603 | cellular amide metabolic process |
| 778 | 6248 | 0.303784825093259 | 1.30097842259494e-43 | < 0.001 | GO:0044237 | cellular metabolic process |
| 514 | 3836 | 0.302975588480242 | 8.23037111047925e-34 | < 0.001 | GO:0016043 | cellular component organization |
| 58 | 389 | 0.30200549951019 | 6.60635248230985e-06 | 0.024 | GO:0051301 | cell division |
| 60 | 405 | 0.298824650792083 | 5.66101867563363e-06 | 0.02 | GO:0005813 | centrosome |
| 453 | 3358 | 0.297472652357805 | 6.71546321567894e-30 | < 0.001 | GO:0046483 | heterocycle metabolic process |
| 457 | 3395 | 0.296812939926031 | 5.60516811109854e-30 | < 0.001 | GO:0006725 | cellular aromatic compound metabolic process |
| 526 | 3998 | 0.293952856550063 | 1.38769082388674e-32 | < 0.001 | GO:0006807 | nitrogen compound metabolic process |
| 132 | 912 | 0.293707992926595 | 7.64034608380786e-11 | < 0.001 | GO:0032403 | protein complex binding |
| 58 | 397 | 0.291480370129831 | 1.23226374127817e-05 | 0.041 | GO:0043900 | regulation of multi-organism process |
| 107 | 739 | 0.291190381589665 | 4.94326210226229e-09 | < 0.001 | GO:0022402 | cell cycle process |
| 771 | 6305 | 0.290230229547624 | 5.85986110725833e-40 | < 0.001 | GO:0044444 | cytoplasmic part |
| 99 | 684 | 0.290177382468302 | 1.90609645235429e-08 | < 0.001 | GO:0019904 | protein domain specific binding |
| 62 | 426 | 0.289778615121839 | 7.09031022107541e-06 | 0.027 | GO:0006886 | intracellular protein transport |
| 871 | 7358 | 0.287822270259692 | 2.58515309026706e-41 | < 0.001 | GO:0008152 | metabolic process |
| 65 | 450 | 0.286090295547268 | 5.53583283686392e-06 | 0.02 | GO:0010564 | regulation of cell cycle process |
| 195 | 1388 | 0.285224953466746 | 2.92426346451795e-14 | < 0.001 | GO:0044877 | macromolecular complex binding |
| 94 | 656 | 0.284456972394892 | 7.06387401449139e-08 | 0.001 | GO:0007049 | cell cycle |
| 290 | 2114 | 0.284140668025122 | 1.43027797728792e-19 | < 0.001 | GO:0000166 | nucleotide binding |
| 290 | 2114 | 0.284140668025122 | 1.43027797728792e-19 | < 0.001 | GO:1901265 | nucleoside phosphate binding |
| 135 | 951 | 0.283821163665819 | 1.75278241750492e-10 | < 0.001 | GO:0033043 | regulation of organelle organization |
| 299 | 2188 | 0.283188250635069 | 6.05971610220169e-20 | < 0.001 | GO:0044267 | cellular protein metabolic process |
| 407 | 3057 | 0.28314523907282 | 2.35863284142126e-25 | < 0.001 | GO:2000112 | regulation of cellular macromolecule biosynthetic process |
| 102 | 717 | 0.281471940294644 | 2.83874794480759e-08 | 0.001 | GO:0016192 | vesicle-mediated transport |
| 597 | 4733 | 0.279618996477169 | 1.97168801231228e-32 | < 0.001 | GO:0031323 | regulation of cellular metabolic process |
| 467 | 3584 | 0.279260437182737 | 2.1260623811583e-27 | < 0.001 | GO:1901360 | organic cyclic compound metabolic process |
| 65 | 456 | 0.279255921754469 | 8.58269395900878e-06 | 0.033 | GO:0031329 | regulation of cellular catabolic process |
| 784 | 6543 | 0.278947968775268 | 1.88187813573583e-37 | < 0.001 | GO:0044238 | primary metabolic process |
| 79 | 557 | 0.277768598856122 | 1.16915023920086e-06 | 0.008 | GO:0051129 | negative regulation of cellular component organization |
| 164 | 1176 | 0.277674004665161 | 7.20171945662278e-12 | < 0.001 | GO:0044430 | cytoskeletal part |
| 309 | 2292 | 0.276989089470516 | 9.88724584328549e-20 | < 0.001 | GO:0034645 | cellular macromolecule biosynthetic process |
| 809 | 6824 | 0.276402057877764 | 2.54243166736962e-37 | < 0.001 | GO:0071704 | organic substance metabolic process |
| 586 | 4657 | 0.276369518992579 | 2.1417005065836e-31 | < 0.001 | GO:0080090 | regulation of primary metabolic process |
| 169 | 1219 | 0.275121580749817 | 5.47293813929024e-12 | < 0.001 | GO:2000113 | negative regulation of cellular macromolecule biosynthetic process |
| 74 | 525 | 0.274144438857691 | 3.18012002533611e-06 | 0.013 | GO:0031400 | negative regulation of protein modification process |
| 431 | 3308 | 0.274063371741301 | 5.09068502612368e-25 | < 0.001 | GO:0031326 | regulation of cellular biosynthetic process |
| 1556 | 16725 | 0.273738699532485 | 7.28214622788467e-26 | < 0.001 | GO:0005575 | cellular component |
| 229 | 1682 | 0.272411492527814 | 4.24514577889766e-15 | < 0.001 | GO:0019899 | enzyme binding |
| 436 | 3360 | 0.272409144312389 | 5.69465794748544e-25 | < 0.001 | GO:0009889 | regulation of biosynthetic process |
| 411 | 3149 | 0.272293637103018 | 6.91191259538263e-24 | < 0.001 | GO:0010556 | regulation of macromolecule biosynthetic process |
| 312 | 2339 | 0.271474594718296 | 3.16013980653872e-19 | < 0.001 | GO:0009059 | macromolecule biosynthetic process |
| 168 | 1220 | 0.271426675182801 | 1.10759138363275e-11 | < 0.001 | GO:0045934 | negative regulation of nucleobase-containing compound metabolic process |
| 436 | 3378 | 0.269309387854651 | 1.74814860609102e-24 | < 0.001 | GO:0051171 | regulation of nitrogen compound metabolic process |
| 182 | 1331 | 0.26926408298585 | 2.62095930156878e-12 | < 0.001 | GO:0033036 | macromolecule localization |
| 370 | 2825 | 0.268757017751433 | 1.40379931947557e-21 | < 0.001 | GO:0051252 | regulation of RNA metabolic process |
| 341 | 2588 | 0.268348840848748 | 3.25511980670492e-20 | < 0.001 | GO:0065010 | extracellular membrane-bounded organelle |
| 406 | 3133 | 0.267508886914279 | 5.80314003106743e-23 | < 0.001 | GO:0019219 | regulation of nucleobase-containing compound metabolic process |
| 861 | 7463 | 0.267374011046418 | 6.3026928847184e-36 | < 0.001 | GO:0005515 | protein binding |
| 342 | 2601 | 0.267310916135486 | 3.96482163773603e-20 | < 0.001 | GO:0043230 | extracellular organelle |
| 340 | 2586 | 0.267007761138424 | 5.34073091089833e-20 | < 0.001 | GO:0070062 | extracellular exosome |
| 173 | 1270 | 0.266236523128076 | 1.31503020693139e-11 | < 0.001 | GO:0005856 | cytoskeleton |
| 341 | 2599 | 0.265972362762961 | 6.49378891223958e-20 | < 0.001 | GO:1903561 | extracellular vesicle |
| 79 | 570 | 0.265850574181754 | 2.87228702605077e-06 | 0.013 | GO:0019901 | protein kinase binding |
| 179 | 1318 | 0.26530866533682 | 7.25742595472605e-12 | < 0.001 | GO:0008104 | protein localization |
| 83 | 601 | 0.264341270174613 | 1.87049618480338e-06 | 0.009 | GO:0007010 | cytoskeleton organization |
| 183 | 1352 | 0.263963642896981 | 5.54178048308321e-12 | < 0.001 | GO:0009890 | negative regulation of biosynthetic process |

*Continued on next page*

| N | X | LOD | P | P adj | attrib ID | attrib name |
|---|---|---|---|---|---|---|
| 355 | 2725 | 0.263781531778374 | 2.94935852064841e-20 | < 0.001 | GO:1903506 | regulation of nucleic acid-templated transcription |
| 108 | 787 | 0.263321309409847 | 7.45325958886638e-08 | 0.001 | GO:0044248 | cellular catabolic process |
| 179 | 1323 | 0.263297338871637 | 1.00258193910575e-11 | < 0.001 | GO:0031327 | negative regulation of cellular biosynthetic process |
| 184 | 1362 | 0.263054105948209 | 5.7015163197515e-12 | < 0.001 | GO:0051172 | negative regulation of nitrogen compound metabolic process |
| 355 | 2731 | 0.262543082718449 | 4.26364324140939e-20 | < 0.001 | GO:2001141 | regulation of RNA biosynthetic process |
| 172 | 1272 | 0.262218934166011 | 2.77328669889121e-11 | < 0.001 | GO:0010558 | negative regulation of macromolecule biosynthetic process |
| 353 | 2718 | 0.261792338785484 | 6.49934076679981e-20 | < 0.001 | GO:0006355 | regulation of transcription, DNA-templated |
| 138 | 1019 | 0.259410015839443 | 2.59518305329458e-09 | < 0.001 | GO:0033554 | cellular response to stress |
| 148 | 1096 | 0.258931749082658 | 8.14046498731766e-10 | < 0.001 | GO:0051253 | negative regulation of RNA metabolic process |
| 77 | 564 | 0.257934289799678 | 6.67499824292365e-06 | 0.024 | GO:0009894 | regulation of catabolic process |
| 642 | 5331 | 0.257735776162497 | 2.60974347245214e-29 | < 0.001 | GO:0019222 | regulation of metabolic process |
| 91 | 670 | 0.256419118330364 | 1.24234768284764e-06 | 0.008 | GO:0044427 | chromosomal part |
| 246 | 1869 | 0.256120720841698 | 1.79289890722067e-14 | < 0.001 | GO:0032774 | RNA biosynthetic process |
| 1423 | 14779 | 0.25587656650761 | 5.03806599507443e-28 | < 0.001 | GO:0003674 | molecular function |
| 244 | 1855 | 0.255540123578109 | 2.51080756046996e-14 | < 0.001 | GO:0006351 | transcription, DNA-templated |
| 244 | 1856 | 0.255248203149733 | 2.66847817120278e-14 | < 0.001 | GO:0097659 | nucleic acid-templated transcription |
| 576 | 4735 | 0.253967667532782 | 9.52374176433698e-27 | < 0.001 | GO:0060255 | regulation of macromolecule metabolic process |
| 85 | 632 | 0.250853877843216 | 4.09684790794260e-06 | 0.02 | GO:0019900 | kinase binding |
| 313 | 2437 | 0.250608203912683 | 7.1186797102455e-17 | < 0.001 | GO:0044271 | cellular nitrogen compound biosynthetic process |
| 311 | 2421 | 0.250465981389568 | 8.94741168795184e-17 | < 0.001 | GO:0036094 | small molecule binding |
| 127 | 954 | 0.249343917996142 | 3.16396957478441e-08 | 0.001 | GO:0015031 | protein transport |
| 139 | 1054 | 0.245611360031055 | 1.24980266150087e-08 | < 0.001 | GO:0045184 | establishment of protein localization |
| 136 | 1032 | 0.244938154710436 | 1.89048340294634e-08 | < 0.001 | GO:0045892 | negative regulation of transcription, DNA-templated |
| 247 | 1919 | 0.244127851026341 | 1.94581251219982e-13 | < 0.001 | GO:0006464 | cellular protein modification process |
| 247 | 1919 | 0.244127851026341 | 1.94581251219982e-13 | < 0.001 | GO:0036211 | protein modification process |
| 98 | 740 | 0.243979265153827 | 1.52975341124885e-06 | 0.008 | GO:0051726 | regulation of cell cycle |
| 137 | 1043 | 0.243324212979641 | 2.04792058942851e-08 | < 0.001 | GO:1903507 | negative regulation of nucleic acid-templated transcription |
| 165 | 1264 | 0.242857348654721 | 1.06022135756955e-09 | < 0.001 | GO:0005829 | cytosol |
| 365 | 2924 | 0.240940621557227 | 9.20190895259982e-18 | < 0.001 | GO:0019538 | protein metabolic process |
| 138 | 1056 | 0.240750048061978 | 2.4897061988985e-08 | < 0.001 | GO:1902679 | negative regulation of RNA biosynthetic process |
| 265 | 2082 | 0.240067687620545 | 8.10224503729758e-14 | < 0.001 | GO:0031324 | negative regulation of cellular metabolic process |
| 129 | 987 | 0.239958987834854 | 7.18373159702484e-08 | 0.001 | GO:0098772 | molecular function regulator |
| 257 | 2023 | 0.238139836023954 | 2.56682953323606e-13 | < 0.001 | GO:0043412 | macromolecule modification |
| 420 | 3422 | 0.238051217289703 | 2.71930433695097e-19 | < 0.001 | GO:0010468 | regulation of gene expression |
| 88 | 672 | 0.237371430343 | 8.1099649583759e-06 | 0.033 | GO:0044429 | mitochondrial part |
| 100 | 766 | 0.236758033864131 | 2.27891770588704e-06 | 0.011 | GO:0005622 | intracellular |
| 258 | 2045 | 0.234483431973348 | 4.99154778644248e-13 | < 0.001 | GO:0032268 | regulation of cellular protein metabolic process |
| 185 | 1447 | 0.233904598413175 | 5.14875010599996e-10 | < 0.001 | GO:0031399 | regulation of protein modification process |
| 408 | 3344 | 0.233147563218257 | 3.0831915124642e-18 | < 0.001 | GO:0031982 | vesicle |
| 185 | 1454 | 0.231348506477917 | 7.58064282261848e-10 | < 0.001 | GO:0022607 | cellular component assembly |
| 115 | 893 | 0.231002982282326 | 7.91282007664003e-07 | 0.006 | GO:0006508 | proteolysis |
| 259 | 2073 | 0.229305206034151 | 1.31929407867801e-12 | < 0.001 | GO:0034654 | nucleobase-containing compound biosynthetic process |
| 369 | 3020 | 0.229304332995061 | 1.7600139076995e-16 | < 0.001 | GO:0031988 | membrane-bounded vesicle |
| 275 | 2215 | 0.227593525268897 | 4.83283619881626e-13 | < 0.001 | GO:0051246 | regulation of protein metabolic process |
| 121 | 951 | 0.225280639080287 | 7.6238115357935e-07 | 0.005 | GO:1901575 | organic substance catabolic process |
| 287 | 2340 | 0.222214443042816 | 5.80645346610832e-13 | < 0.001 | GO:0009892 | negative regulation of metabolic process |
| 141 | 1120 | 0.221557226632652 | 1.70568430987916e-07 | 0.002 | GO:0005794 | Golgi apparatus |
| 262 | 2143 | 0.217833008935442 | 1.02906935442488e-11 | < 0.001 | GO:0019438 | aromatic compound biosynthetic process |
| 261 | 2135 | 0.217683839646184 | 1.14608268217442e-11 | < 0.001 | GO:0018130 | heterocycle biosynthetic process |
| 204 | 1653 | 0.217039814330807 | 1.26996288879982e-09 | < 0.001 | GO:0035639 | purine ribonucleoside triphosphate binding |
| 172 | 1390 | 0.215388470633394 | 2.36066985332072e-08 | < 0.001 | GO:0010629 | negative regulation of gene expression |
| 257 | 2110 | 0.215282476848721 | 2.49582539769764e-11 | < 0.001 | GO:0010605 | negative regulation of macromolecule metabolic process |
| 143 | 1152 | 0.214399334515797 | 3.20244274291729e-07 | 0.004 | GO:0032270 | positive regulation of cellular protein metabolic process |
| 116 | 931 | 0.214030166768131 | 3.57743604588394e-06 | 0.013 | GO:0043085 | positive regulation of catalytic activity |
| 209 | 1709 | 0.212678742442493 | 1.68498212749882e-09 | < 0.001 | GO:0017076 | purine nucleotide binding |
| 204 | 1667 | 0.212552728632928 | 2.55909104963045e-09 | < 0.001 | GO:0032550 | purine ribonucleoside binding |
| 204 | 1669 | 0.211914960168873 | 2.82457373520506e-09 | < 0.001 | GO:0032549 | ribonucleoside binding |
| 204 | 1670 | 0.211596377673768 | 2.96708735842337e-09 | < 0.001 | GO:0001883 | purine nucleoside binding |
| 140 | 1134 | 0.211298568835294 | 5.83934932522019e-07 | 0.004 | GO:0009056 | catabolic process |
| 355 | 2996 | 0.210375053579688 | 6.94893778484177e-14 | < 0.001 | GO:0044249 | cellular biosynthetic process |
| 165 | 1347 | 0.209427863611025 | 9.15272128355743e-08 | 0.001 | GO:0005524 | ATP binding |
| 204 | 1677 | 0.209371906954504 | 4.17779832755258e-09 | < 0.001 | GO:0001882 | nucleoside binding |
| 1437 | 15496 | 0.209247325575838 | 9.38106170218584e-19 | < 0.001 | GO:0008150 | biological_process |
| 120 | 974 | 0.208551215379492 | 4.2045576791455e-06 | 0.02 | GO:0065003 | macromolecular complex assembly |
| 206 | 1699 | 0.207818234076543 | 4.54581650847298e-09 | < 0.001 | GO:0032555 | purine ribonucleotide binding |
| 150 | 1227 | 0.207050705447106 | 4.10399750803945e-07 | 0.004 | GO:0042802 | identical protein binding |
| 134 | 1095 | 0.206215079080603 | 1.64923849966414e-06 | 0.009 | GO:0071822 | protein complex subunit organization |
| 207 | 1713 | 0.206122619165961 | 5.46766716056327e-09 | < 0.001 | GO:0032553 | ribonucleotide binding |
| 169 | 1393 | 0.204736601369246 | 1.20674107194648e-07 | 0.001 | GO:0030554 | adenyl nucleotide binding |
| 247 | 2068 | 0.203687618866109 | 4.47219989659628e-10 | < 0.001 | GO:0051128 | regulation of cellular component organization |
| 167 | 1379 | 0.203612954019887 | 1.62219310666521e-07 | 0.002 | GO:0031090 | organelle membrane |
| 151 | 1248 | 0.201754004347399 | 6.93243412385515e-07 | 0.004 | GO:0051247 | positive regulation of protein metabolic process |
| 359 | 3079 | 0.201702441197661 | 4.62818155823585e-13 | < 0.001 | GO:1901576 | organic substance biosynthetic process |
| 167 | 1385 | 0.201336844964681 | 2.15346318843317e-07 | 0.003 | GO:0032559 | adenyl ribonucleotide binding |
| 265 | 2240 | 0.20025573546515 | 2.44342472984665e-10 | < 0.001 | GO:1901362 | organic cyclic compound biosynthetic process |
| 134 | 1111 | 0.198699094323194 | 3.53846079631159e-06 | 0.013 | GO:0030054 | cell junction |
| 485 | 4289 | 0.197683677705686 | 1.57467023730153e-15 | < 0.001 | GO:0048522 | positive regulation of cellular process |
| 363 | 3141 | 0.197110704761263 | 1.11978765363476e-12 | < 0.001 | GO:0009058 | biosynthetic process |
| 281 | 2398 | 0.196606097500472 | 1.7361796172996e-10 | < 0.001 | GO:0043168 | anion binding |
| 438 | 3858 | 0.194816378083758 | 3.65037940767421e-14 | < 0.001 | GO:0048523 | negative regulation of cellular process |
| 241 | 2055 | 0.193257945192023 | 4.06103514258298e-09 | < 0.001 | GO:0006950 | response to stress |

*Continued on next page*

Supplementary Table 5 – *Continued from previous page*

| N | X | LOD | P | P adj | attrib ID | attrib name |
|---|---|---|---|---|---|---|
| 184 | 1558 | 0.191902936325537 | 2.12645650214527e-07 | 0.003 | GO:0050790 | regulation of catalytic activity |
| 237 | 2042 | 0.187303139606244 | 1.3870495901529e-08 | $< 0.001$ | GO:0065009 | regulation of molecular function |
| 528 | 4800 | 0.185808101887747 | 9.43835180637959e-15 | $< 0.001$ | GO:0048518 | positive regulation of biological process |
| 387 | 3443 | 0.183727318929724 | 8.07563944960964e-12 | $< 0.001$ | GO:0051179 | localization |
| 234 | 2037 | 0.181511827821309 | 4.14340388565047e-08 | 0.001 | GO:0097367 | carbohydrate derivative binding |
| 463 | 4200 | 0.180111742521721 | 6.82029573178409e-13 | $< 0.001$ | GO:0048519 | negative regulation of biological process |
| 548 | 5062 | 0.17817149210099 | 4.74173483179147e-14 | $< 0.001$ | GO:0043167 | ion binding |
| 383 | 3443 | 0.177385461539469 | 4.37662169813125e-11 | $< 0.001$ | GO:0044421 | extracellular region part |
| 331 | 2950 | 0.177345716017763 | 5.10724790352309e-10 | $< 0.001$ | GO:0009893 | positive regulation of metabolic process |
| 186 | 1631 | 0.173646244990099 | 1.89526819902828e-06 | 0.009 | GO:0005739 | mitochondrion |
| 804 | 7864 | 0.170508510841268 | 1.21826344562588e-15 | $< 0.001$ | GO:0044763 | single-organism cellular process |
| 283 | 2536 | 0.170039037443341 | 2.0912007504424e-08 | $< 0.001$ | GO:0031325 | positive regulation of cellular metabolic process |
| 344 | 3116 | 0.169434993126536 | 1.42348344409549e-09 | $< 0.001$ | GO:0051234 | establishment of localization |
| 204 | 1809 | 0.169151926540129 | 1.25911864015809e-06 | 0.008 | GO:0003677 | DNA binding |
| 277 | 2511 | 0.163228913450447 | 8.79526317235677e-08 | 0.001 | GO:0010604 | positive regulation of macromolecule metabolic process |
| 326 | 2979 | 0.162927401730422 | 1.1137681740031e-08 | $< 0.001$ | GO:0006810 | transport |
| 174 | 1556 | 0.162434661108913 | 1.31843928950383e-05 | 0.045 | GO:0006357 | regulation of transcription from RNA polymerase II promoter |
| 215 | 1958 | 0.155856044324931 | 4.1301598910317e-06 | 0.02 | GO:0051716 | cellular response to stimulus |
| 233 | 2139 | 0.152921951654374 | 2.79573206938709e-06 | 0.013 | GO:0016787 | hydrolase activity |
| 910 | 9420 | 0.140614011973023 | 1.4515934228624e-11 | $< 0.001$ | GO:0050794 | regulation of cellular process |
| 948 | 9917 | 0.136723525222993 | 4.43660520702556e-11 | $< 0.001$ | GO:0050789 | regulation of biological process |
| 501 | 4928 | 0.13483794931965 | 1.81645476616066e-08 | $< 0.001$ | GO:0003824 | catalytic activity |
| 414 | 4076 | 0.127410482165843 | 5.96421399016038e-07 | 0.004 | GO:0044767 | single-organism developmental process |
| 970 | 10304 | 0.126368027983229 | 9.99252846357788e-10 | $< 0.001$ | GO:0065007 | biological regulation |
| 334 | 3264 | 0.12593387303247 | 5.31070724510026e-06 | 0.02 | GO:0046872 | metal ion binding |
| 432 | 4277 | 0.125468107133004 | 5.77748110116313e-07 | 0.004 | GO:0032502 | developmental process |
| 339 | 3342 | 0.121341005835235 | 9.60828469425636e-06 | 0.036 | GO:0043169 | cation binding |

**Supplementary Table 6: List of primers used.**

| primer | sequence |
| --- | --- |
| mL32/5′ | AACCCAGAGGCATTGACAAC |
| mL32/3′ | ATTGTGGACCAGGAACTTGC |
| mICAM/5′ | CACGCTACCTCTGCTCCTG |
| mICAM/3′ | AAGGCTTCTCTGGGATGGAT |
| Taqman mcircRasGEF1B/5′ | CCGGGACGAGAGAATGATGA |
| Taqman mcircRasGEF1B/3′ | GGACTGGTAGAGGTTTCGGTTG |
| Taqman mcircRasGEF1B probe | CAGTGGCGAGGAGGA |
| Control ASO | 5′mC*mC*mA*mG*mU*mG*G*C*G*A*G*G*A*G*G*A*A*A*mG*mU*mA*mU*mG*mC3′ |
| mcircRasGEF1B ASO I | 5′mG*mC*mA*mU*mA*mC*T*T*T*C*C*T*C*C*T*C*G*C*mC*mA*mC*mU*mG*mG3′ |
| mcircRasGEF1B ASO II | 5′mC*mU*mU*mU*mC*mC*T*C*C*T*C*G*C*C*A*C*T*G*mG*mC*mC*mA*mU*mC3′ |
